# Copper-catalyzed multicomponent reaction of β-trifluoromethyl β-diazo esters enabling the synthesis of β-trifluoromethyl *N*,*N*-diacyl-β-amino esters

**DOI:** 10.3762/bjoc.20.21

**Published:** 2024-02-02

**Authors:** Youlong Du, Haibo Mei, Ata Makarem, Ramin Javahershenas, Vadim A Soloshonok, Jianlin Han

**Affiliations:** 1 Jiangsu Co-Innovation Center of Efficient Processing and Utilization of Forest Resources, College of Chemical Engineering, Nanjing Forestry University, Nanjing 210037, China; 2 Department of Chemistry, University of Hamburg, Martin-Luther-King-Platz 6, 20146 Hamburg, Germany; 3 Department of Organic Chemistry, Faculty of Chemistry, Urmia University, Urmia, Iran; 4 Department of Organic Chemistry I, Faculty of Chemistry, University of the Basque Country (UPV/EHU), Paseo Manuel Lardizábal 3, San Sebastián, 20018, Spain; 5 IKERBASQUE, Basque Foundation for Science, Alameda Urquijo 36-5, Plaza Bizkaia, 48011 Bilbao, Spain

**Keywords:** β-carbonyl diazo, copper catalyst, fluoroalkyl diazo, Mumm rearrangement, unsymmetrical β-diacylamino esters

## Abstract

An efficient multicomponent reaction of newly designed β-trifluoromethyl β-diazo esters, acetonitrile, and carboxylic acids via an interrupted esterification process under copper-catalyzed conditions has been developed, which affords various unsymmetrical β-trifluoromethyl *N*,*N*-diacyl-β-amino esters in good to excellent yields. The reaction features mild conditions, a wide scope of β-amino esters and carboxylic acids, and also applicability to large-scale synthesis, thus providing an efficient way for the synthesis of β-trifluoromethyl β-diacylamino esters. Furthermore, this reaction represents the first example of a Mumm rearrangement of β-trifluoromethyl β-diazo esters.

## Introduction

The insertion of fluorine-containing components frequently confers desirable physical and biological properties to organic molecules, and the development of fluorine-containing drugs is an important field of research [1–9]. It is estimated that 30% of drugs and over 50% of agricultural chemicals contain at least one fluorine atom, among which architectural motifs containing fluorine and amino acid residues are a fast-growing segment of modern pharmaceuticals [10–13].

Fluoroalkyldiazo compounds belong to the most versatile and valuable reagents in organic synthesis, as they can be used as diazo intermediates or carbene precursors for the rapid construction of complex molecules along with the introduction of fluoroalkyl groups [14–16]. Although the reaction of trifluorodiazoethane [17–27] as well as α-diazo esters [28–30] have been widely explored, β-trifluoromethyl β-diazo esters have been less investigated, mainly due to the instability of such structures. Therefore, methods for the synthesis of β-trifluoromethyl β-diazo esters and their applications in organic synthesis are needed but remain challenging.

**Scheme 1:**
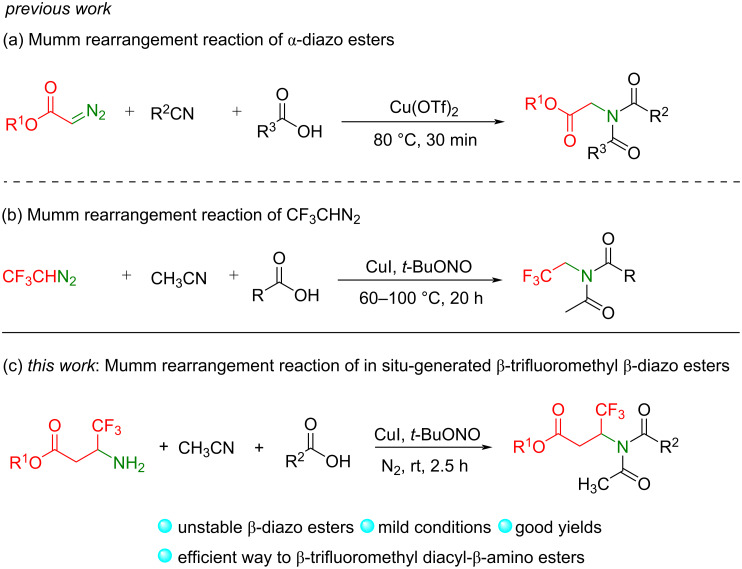
Mumm-type rearrangement of diazo compounds.

On the other hand, several interesting transformations of nitrile ylides from diazo compounds have been developed in the past years [31–38]. In particular, acylglycine esters could be easily constructed with ester-containing diazo compounds as the starting materials. For example, Wan and co-workers developed a cascade reaction of α-diazo esters, nitriles, and carboxylic acids via the generation of nitrile ylides and Mumm rearrangement affording unsymmetric diacyl α-amino acid esters as products (Scheme 1a) [39]. In 2017, Zhang, Hu, and co-workers developed a Cu-catalyzed reaction of CF_3_CHN_2_ with carboxylic acids and acetonitrile via a similar process to afford a series of *N*-trifluoroethylimides (Scheme 1b) [40–41]. Inspired by these elegant works [31–41] and based on our continuous interest in reactions of fluoroalkyldiazo compounds [42–49], we sought to develop reactions of the unexplored β-trifluoromethyl β-diazo esters. We hypothesized that nitrile ylides, in situ generated from nitriles and β-trifluoromethyl β-amino esters, could also react with carboxylic acids to give nitriliums, which then could undergo a Mumm rearrangement to provide unsymmetrical β-trifluoromethyl diacyl-β-amino esters as products (Scheme 1c). Herein, we report our results on the design of β-trifluoromethyl β-diazo esters and their application in a three-component reaction with nitriles and carboxylic acids under mild conditions. A variety of unnatural unsymmetrical β-trifluoromethyl diacyl-β-amino esters were obtained in good yields, which are useful synthetic scaffolds [50–52] but difficult to obtain by other methods [53–57]. This work is the first example of the reaction of β-trifluoromethyl β-diazo esters, which enriches the studied content of fluoroalkyl diazo compounds.

## Results and Discussion

Due to the instability of β-carbonyl diazo compounds and the occurrence of possible side reactions [58–61], screening of reaction conditions to optimize this conversion and inhibit the occurrence of side reactions was carried out with benzyl 3-amino-4,4,4-trifluorobutanoate (**1a**) and benzoic acid (**3a**) as model substrates. The initial reaction of amine **1a** and acid **3a** in acetonitrile in the presence of diazotization reagent *tert*-butyl nitrite with CuI (10 mol %) as catalysts for 2.5 h at room temperature proceeded to afford the desired unsymmetrical β-trifluoromethyl diacyl-β-amino ester **4a** in 54% yield (Table 1, entry 1). The loading amount of catalyst CuI plays a crucial role in the formation of the desired product **4a**. Increasing the loading amount of CuI, the yield could be raised to 66% when 20 mol % of CuI was used as catalyst (Table 1, entries 2 and 3). However, further increasing the amount of the catalyst led to an obvious decrease in the yield of product **4a** (Table 1, entries 4 and 5). Variation on the reaction temperature also afforded the corresponding product **4a** but failed to bring any improvement on the reaction outcome (Table 1, entries 6 and 7). Further optimization of the reaction conditions focused on the variation of the amounts of amine **1a** and *tert*-butyl nitrite (Table 1, entries 8–12). Considering the instability of the diazo structure generated from amine **1a**, we increased the amount of amine **1a** and *tert*-butyl nitrite to 4 equivalents. Pleasingly, the yield of product **4a** was further increased to 74% (Table 1, entry 12). Furthermore, we optimized the reaction time and found that shortening the reaction time resulted in a decreased yield (Table 1, entry 13). Increasing the reaction time to 3 h also did not lead to any better result mainly due to the decomposition of product **4a** (Table 1, entry 14).

**Table 1:** Optimization of reaction conditions.^a^

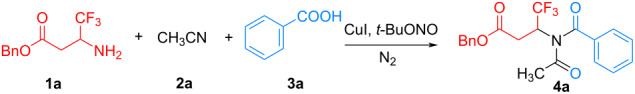						

Entry	**1a** (equiv)	CuI (mol %)	*t*-BuONO (equiv)	*T* (°C)	Time (h)	Yield^b^ (%)

1	2	10	2	rt	2.5	54
2	2	15	2	rt	2.5	57
3	2	20	2	rt	2.5	66
4	2	30	2	rt	2.5	32
5	2	40	2	rt	2.5	trace
6	2	20	2	0	2.5	20
7	2	20	2	60	2.5	38
8	2	20	1	rt	2.5	38
9	2	20	3	rt	2.5	41
10	1	20	2	rt	2.5	27
11	3	20	2	rt	2.5	43
12	4	20	4	rt	2.5	74
13	4	20	4	rt	1.5	43
14	4	20	4	rt	3	60

^a^Reaction conditions: amine **1a** (0.4 mmol), benzoic acid **3a** (0.1 mmol), CuI (20 mol %), *t*-BuONO (0.4 mmol) and CH_3_CN (2 mL) under nitrogen atmosphere. ^b^Isolated yield based on acid **3a**.

**Scheme 2:**
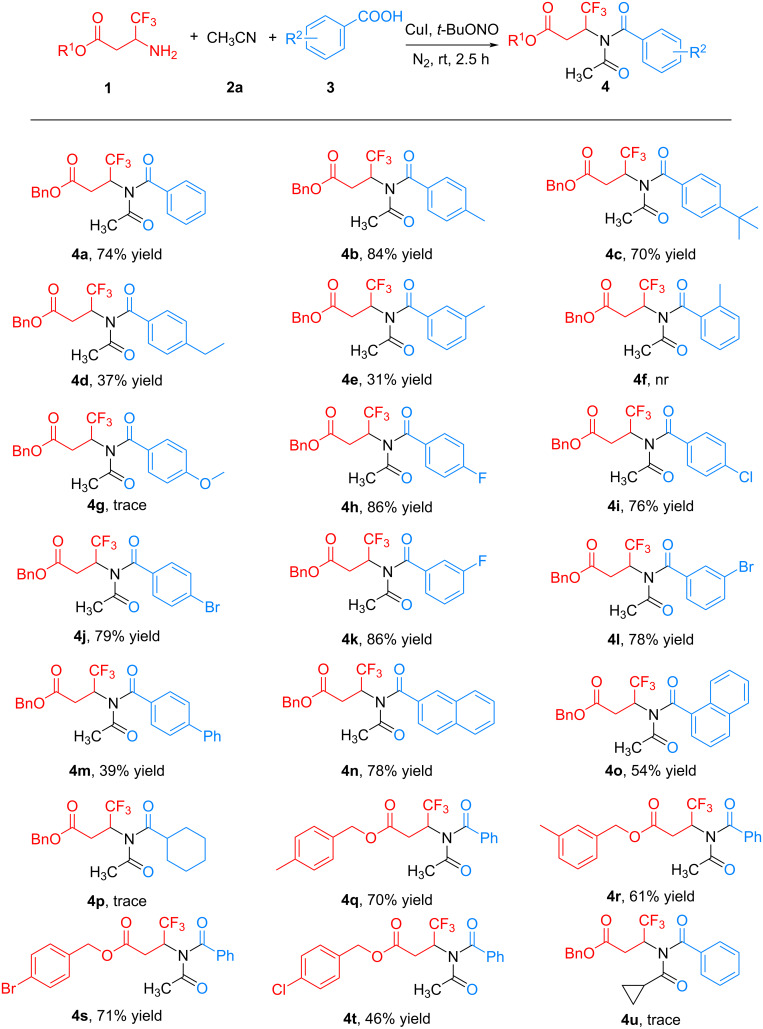
Substrate scope study of this Cu-catalyzed reaction.

With the optimized reaction conditions in hand, we next evaluated the substrate scope by using a variety of structurally diverse carboxylic acids **3** to react with β-trifluoromethyl β-amino esters **1**. As shown in Scheme 2, all the substituted benzoic acids **3** tested were well tolerated in this reaction, and the corresponding product **4** was successfully prepared at moderate to excellent yields (**4a**–**e**, **4h**–**m**, 31–86% yields). The benzoic acids featuring a wide range of functional groups, including alkyl (**4a**–**e**), halogen (**4h**–**l**), and phenyl (**4m**), were all suitable substrates for this reaction. However, the benzoic acid with *ortho*-substituent did not afford the expected product (**4f**) mainly due to the steric hindrance effect. Notably, substrates with electron-withdrawing groups **(4h**–**l**, 76–86% yields) provided better chemical yields in this reaction compared with those containing electron-donating groups (**4b**–**e**, 31–84% yields). For the case with a strong electron-donating group (methoxy, **3g**) only traces of **4g** were produced. Besides benzoic acid, the current Cu-catalyzed reaction was also applicable to other aromatic acid substrates. Using 2-naphthoic and 1-naphthoic acid as substrates, the corresponding products **4n** and **4o** were produced well with yields of 78% and 54%, respectively. Unfortunately, the tested aliphatic acid, such as cyclohexanecarboxylic acid, did not work in the system to produce the expected product (**4p**). In addition, the β-trifluoromethyl β-amino benzyl ester substrates **1** with different ester groups were tried to react with benzoic acid (**3a**) to further extend the substrate range. To our delight, both the amines with electron-donating groups (**4q** and **4r**) and electron-withdrawing groups (**4s** and **4t**) could generate the target products with moderate to good yields (46–71%). We also tried another nitrile substrate, such as cyclopropyl acetonitrile, which yielded only very small amounts of the expected product (**4u**).

**Scheme 3:**
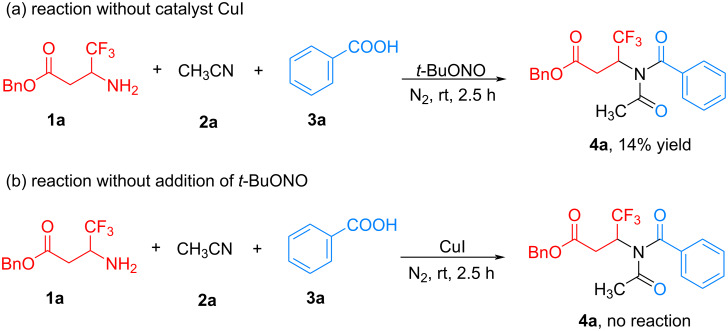
Control experiments.

To gain insight into the mechanism of this reaction, several control experiments were performed. First, the reaction was conducted under the optimized conditions without the addition of CuI. The conversion of the starting substrates to the desired product **4a** was decreased and only 14% yield of **4a** was obtained (Scheme 3a). However, as shown in entry 1 of Table 1, 54% yield product **4a** was produced by this reaction in the presence of 10 mol % CuI. These results demonstrate that copper catalysis plays a crucial role in the generation of the desired product **4a**. Moreover, we performed this reaction without the addition of *tert*-butyl nitrite (Scheme 3b). The expected three-component tandem reaction did not occur, and the target **4a** was not observed with almost all of the starting amine **1a** remaining. This result indicates the reaction proceeds through the diazo intermediate.

**Scheme 4:**
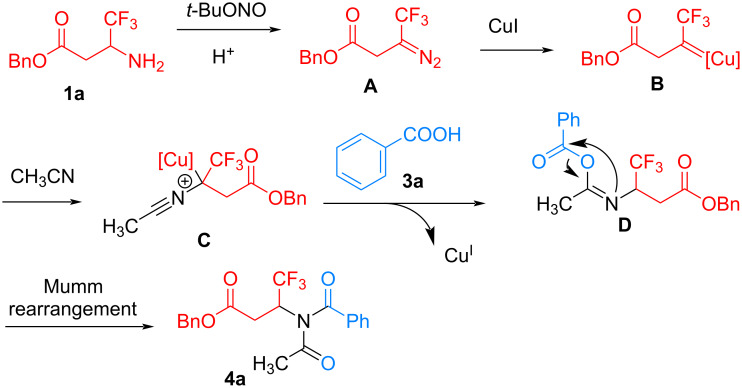
Proposed reaction mechanism.

According to the above experimental results and literature reports [39–41,58–59], a possible mechanism for this Cu-catalyzed reaction of β-trifluoromethyl β-amino esters was proposed in Scheme 4. Initially, β-trifluoromethyl β-amino ester **1a** reacts with *tert*-butyl nitrite to form trifluoromethylated β-carbonyl diazo intermediate **A**. Then, the diazo intermediate **A** reacts with the copper catalyst generating the Cu-carbene intermediate **B**, which undergoes nucleophilic attack by acetonitrile to form the intermediate **C**. Subsequently, nucleophilic addition of benzoic acid to intermediate **C** affords the acetimidic anhydride **D** with the release of Cu^I^ catalyst for the next catalytic cycle. Finally, the acetimidic anhydride **D** undergoes a Mumm rearrangement to furnish the desired β-trifluoromethyl diacylamino ester **4a**.

**Scheme 5:**
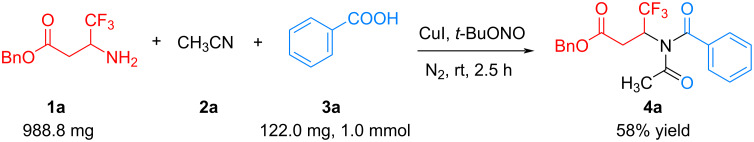
Scale-up synthesis.

The final goal of this work is the examination of the scale-up applicability of this three-component tandem reaction (Scheme 5). To our delight, the reaction also proceeded smoothly when the amount of β-trifluoromethyl β-amino ester **1a** was increased ten-fold to 988.8 mg. The corresponding β-trifluoromethyl diacylamino ester **4a** was obtained in 58% chemical yield. This result indicates the wide synthesis utility of the reactions reported in this work.

## Conclusion

In summary, a series of new β-trifluoromethyl β-diazo esters have been designed, which are applied for the first time in a cascade reaction through an interrupted esterification with nitrile ylides as the key intermediates under copper-catalysis conditions. Varieties of unsymmetric trifluoromethyl diacyl β-amino esters can be easily constructed with good chemical yields. The reaction is conducted under mild conditions and shows good applicability to different series of substrates, which provides an efficient way for the preparation of unsymmetric trifluoromethyl diacyl β-amino esters.

## Experimental

### General procedure for copper-catalyzed multicomponent reaction of β-amino esters

Into a flask were added amines **1** (0.4 mmol), acids **3** (0.1 mmol), CuI (20 mol %), and CH_3_CN (2 mL). Then, the mixture was stirred at room temperature under a nitrogen atmosphere and *t*-BuONO (0.4 mmol) was added dropwise. Stirring was continued at room temperature for 2.5 h and the solvent was removed in vacuum. Products **4** were purified on a TLC plate of 20 cm × 20 cm using petroleum ether/ethyl acetate 7:1 (v/v) as eluent.

## Supporting Information

File 1 Experimental details and spectral data.

## Data Availability

The data that supports the findings of this study is available from the corresponding author upon reasonable request.
