# Supplementary material for: Copper-catalyzed multicomponent reaction of β-trifluoromethyl β-diazo esters enabling the synthesis of β-trifluoromethyl N,N-diacyl-β-amino esters
Source: Beilstein J Org Chem. 2024 Feb 2;20:212–9. doi: 10.3762/bjoc.20.21 (PMC10840549; doi:10.3762/bjoc.20.21)

BEILSTEIN JOURNAL OF ORGANIC CHEMISTRY

# Supporting Information

for

# Copper-catalyzed multicomponent reaction of β-trifluoromethyl β-diazo esters enabling the synthesis of β-trifluoromethyl *N*,*N*-diacyl-β-amino esters

Youlong Du, Haibo Mei, Ata Makarem, Ramin Javahershenas, Vadim A. Soloshonok and Jianlin Han

*Beilstein J. Org. Chem.* **2024,** *20,* 212–219. doi:10.3762/bjoc.20.21

# Experimental details and spectral data

License and Terms: This is a supporting information file under the terms of the Creative Commons Attribution License (https://creativecommons.org/licenses/by/4.0). Please note that the reuse, redistribution and reproduction in particular requires that the author(s) and source are credited and that individual graphics may be subject to special legal provisions.
The license is subject to the *Beilstein Journal of Organic Chemistry* terms and conditions: (https://www.beilstein-journals.org/bjoc/terms)

## Table of contents

**1. General information ………………………………………………………….. S2**

**2. General procedure for copper-catalyzed reaction of β-diazo esters..………. S2**

**3. Large-scale synthesis…………………………………..………………..S3**

**4. Characterization data of compounds 4..……………………….……………S3**

**5. $^{1}$H, $^{13}$C, $^{19}$F NMR spectra for compounds 4…..………………..…...………..S11**

# 1. General information

All the commercial reagents including solvents were used directly without further purification. Amines **1** were synthesized according to the literature.[1] All the experiments were monitored by thin layer chromatography (TLC) with UV light. The TLC employed 0.25 mm silica gel coated on glass plates. Purification of products was carried out by silica gel 60 F-254 TLC plates of 20 cm × 20 cm and column chromatography with silica gel 60 (300–400 mesh). NMR spectra were recorded on Bruker 400 MHz spectrometers. High-resolution mass spectra (HRMS) were measured on an Agilent 6210 ESI/TOF MS instrument.

1. Y. Dai, C. Xie, H. Mei, J. Han, V. A. Soloshonok, Y. Pan, *Tetrahedron* **2015**, *71*, 9550- 9556;

# 2. General procedure for the Cu-catalyzed reaction of β-diazo esters

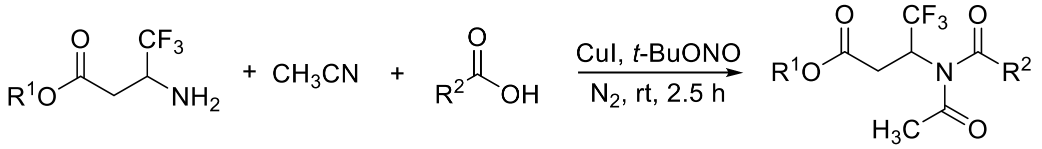


**1** **2a** **3** **4**

Into the flask were added amines **1** (0.4 mmol), acids **3** (0.1 mmol), CuI (20 mol %), and $CH_3CN$ (2 mL). Then, the mixture was stirred at room temperature under nitrogen atmosphere and *t*-BuONO (0.4 mmol) was added dropwise. Stirring was continued at room temperature for 2.5 h, solvent was removed in vacuum. The product **4** was purified by TLC plate of 20 cm × 20 cm using petroleum ether/ethyl acetate 7:1 (v/v) as eluent.

## 3. Large-scale synthesis

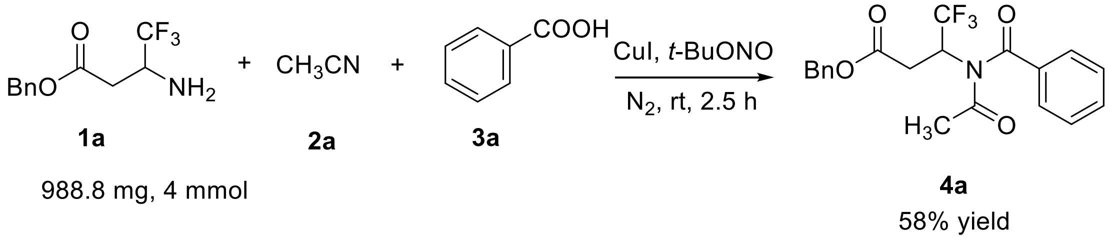


Amine **1a** (4 mmol), acid **3a** (1 mmol), CuI (0.2 mmol), $CH_3CN$ (20 mL) and *t*-BuONO (4 mmol) were added sequentially in a 100 mL flask under nitrogen atmosphere. After stirring the mixture for 2.5 h at room temperature, the solvent was removed in vacuum. The product **4a** was purified by column chromatography using petroleum ether/ethyl acetate 5:1 (v/v) as eluent, and the pure product was isolated in 228.1 mg (58% yield).

## 4. Characterization data of compounds 4

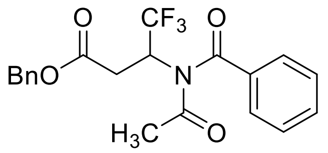

Compound **4a**: 29.1 mg, 74% yield, colorless oil. $^1H$ NMR (400 MHz, $CDCl_3$): δ = 7.73-7.67 (m, 2H), 7.66-7.57 (m, 1H), 7.52-7.44 (m, 2H), 7.41-7.30 (m, 5H), 5.74-5.60 (m, 1H), 5.24-5.10 (m, 2H), 3.64 (dd, *J* = 11.28, 17.04 Hz, 1H), 3.06-2.92 (m, 1H), 1.85 (s, 3H). $^{13}C$ NMR (100 MHz, $CDCl_3$): δ = 173.4, 173.0, 169.3, 135.7, 135.3, 133.6, 129.2, 128.7, 128.6, 128.5, 128.4, 125.8 (q, *J* = 281.0 Hz), 67.0, 54.0 (q, *J* = 32.5 Hz), 31.6, 27.0. $^{19}F\{^1H\}$ NMR (376 MHz, $CDCl_3$): δ = -71.2 (s). HRMS (ESI) m/z: $[M+H]^+$ calcd for $C_{20}H_{19}F_3NO_4^+$ 394.1261, found 394.1264.

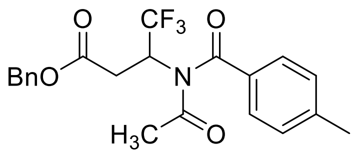

Compound **4b**: 34.2 mg, 84% yield, colorless oil. $^1H$ NMR (400 MHz, $CDCl_3$): δ = 7.64-7.57 (m,

2H), 7.44-7.30 (m, 6H), 7.27-7.23 (m, 1H), 5.77-5.60 (m, 1H), 5.23-5.09 (m, 2H), 3.61 (dd, $J$ = 11.24, 17.20 Hz, 1H), 3.02 (dd, $J$ = 4.00, 17.20 Hz, 1H), 2.45 (s, 3H), 1.85 (s, 3H). $^{13}$C NMR (100 MHz, $CDCl_3$): δ = 173.3, 172.9, 169.3, 144.9, 135.3, 132.8, 129.9, 129.4, 128.6, 128.5, 128.4, 125.8 (q, $J$ = 281.1 Hz), 67.0, 54.1 (q, $J$ = 35.4 Hz), 31.7, 26.9, 21.8. $^{19}$F{$^1$H} NMR (376 MHz, $CDCl_3$): δ = -71.3 (s). HRMS (ESI) m/z: $[M+H]^+$ calcd for $C_{21}H_{21}F_3NO_4^+$ 408.1417, found 408.1414.

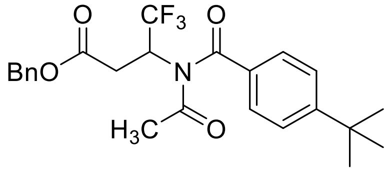


Compound **4c**: 31.4 mg, 70% yield, colorless oil. $^1$H NMR (400 MHz, $CDCl_3$): δ = 7.68-7.59 (m, 2H), 7.52-7.44 (m, 2H), 7.42-7.30 (m, 5H), 5.76-5.61 (m, 1H), 5.24-5.09 (m, 2H), 3.62 (dd, $J$ = 11.44, 17.04 Hz, 1H), 3.04-2.94 (m, 1H), 1.86 (s, 3H), 1.36 (s, 9H). $^{13}$C NMR (100 MHz, $CDCl_3$): δ = 173.3, 172.9, 169.3, 157.8, 135.3, 132.7, 129.3, 128.6, 128.4, 128.3, 128.1 (q, $J$ = 225.3 Hz), 126.2, 67.0, 54.2 (q, $J$ = 31.9 Hz), 35.3, 31.7, 31.1, 26.9. $^{19}$F{$^1$H} NMR (376 MHz, $CDCl_3$): δ = -71.2 (s). HRMS (ESI) m/z: $[M+H]^+$ calcd for $C_{24}H_{27}F_3NO_4^+$ 450.1887, found 450.1879.

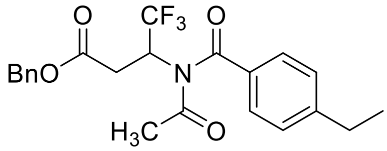


Compound **4d**: 15.6 mg, 37% yield, colorless oil. $^1$H NMR (400 MHz, $CDCl_3$): δ = 7.66-7.58 (m, 2H), 7.43-7.30 (m, 7H), 5.75-5.61 (m, 1H), 5.23-5.09 (m, 2H), 3.61 (dd, $J$ = 11.16, 17.20 Hz, 1H), 3.02 (dd, $J$ = 5.32, 17.20 Hz, 1H), 2.79-2.69 (m, 2H), 1.85 (s, 3H), 1.36-1.23 (m, 3H). $^{13}$C NMR (100 MHz, $CDCl_3$): δ = 173.3, 172.9, 169.3, 150.9, 135.3, 133.0, 129.5, 128.7, 128.6, 128.5, 128.4, 125.8 (q, $J$ = 281.1 Hz), 67.0, 53.8 (q, $J$ = 31.8 Hz), 31.7, 29.0, 26.8, 15.0. $^{19}$F{$^1$H} NMR (376 MHz, $CDCl_3$): δ = -71.3 (s). HRMS (ESI) m/z: $[M+H]^+$ calcd for $C_{22}H_{23}F_3NO_4^+$ 422.1574, found 422.1571.

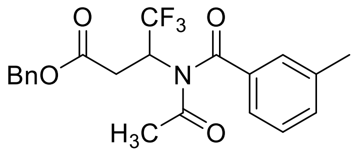

Compound **4e**: 12.6 mg, 31% yield, colorless oil. $^{1}$H NMR (400 MHz, $CDCl_3$): δ = 7.54 (s, 1H), 7.49-7.41 (m, 2H), 7.40-7.31 (m, 6H), 5.75-5.60 (m, 1H), 5.20-5.11 (m, 2H), 3.63 (dd, *J* = 11.12, 17.24 Hz, 1H), 3.02 (dd, *J* = 4.40, 17.24 Hz, 1H), 2.42 (s, 3H), 1.85 (s, 3H). $^{13}$C NMR (100 MHz, $CDCl_3$): δ = 173.6, 173.1, 169.3, 139.3, 135.6, 135.3, 134.4, 129.6, 129.0, 128.6, 128.5, 128.4, 126.3, 125.8 (q, *J* = 281.0 Hz), 67.0, 53.9 (q, *J* = 31.8 Hz), 31.7, 27.0, 21.3. $^{19}$F{$^{1}$H} NMR (376 MHz, $CDCl_3$): δ = -71.2 (s). HRMS (ESI) m/z: $[M+H]^+$ calcd for $C_{21}H_{21}F_3NO_4^+$ 408.1417, found 408.1411.

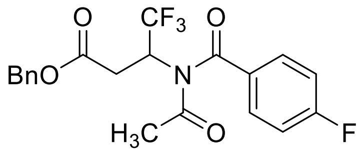

Compound **4h**: 35.3 mg, 86% yield, colorless oil. $^{1}$H NMR (400 MHz, $CDCl_3$): δ = 7.75-7.67 (m, 2H), 7.42-7.31 (m, 5H), 7.20-7.10 (m, 2H), 5.69-5.55 (m, 1H), 5.22-5.10 (m, 2H), 3.61 (dd, *J* = 11.32, 17.28 Hz, 1H), 3.01 (dd, *J* = 4.24, 17.32 Hz, 1H), 1.88 (s, 3H). $^{13}$C NMR (100 MHz, $CDCl_3$): δ = 172.7, 172.2, 169.3, 167.2 (d, *J* = 255.3 Hz), 135.2, 131.9 (d, *J* = 9.3 Hz), 131.7, 128.6, 128.5, 128.4, 125.7 (q, *J* = 281.1 Hz), 116.6 (d, *J* = 22.2 Hz), 67.1, 54.1 (q, *J* = 31.4 Hz), 31.6, 26.8. $^{19}$F{$^{1}$H} NMR (376 MHz, $CDCl_3$): δ = -71.3 (s), -103.3 (s). HRMS (ESI) m/z: $[M+H]^+$ calcd for $C_{20}H_{18}F_4NO_4^+$ 412.1166, found 412.1162.

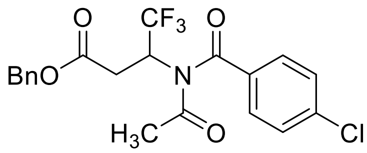

Compound **4i**: 32.5 mg, 76% yield, colorless oil. $^{1}$H NMR (400 MHz, $CDCl_3$): δ = 7.67-7.56 (m, 2H), 7.48-7.41 (m, 2H), 7.40-7.29 (m, 5H), 5.68-5.51 (m, 1H), 5.22-5.09 (m, 2H), 3.62 (dd, *J* = 11.92, 17.24 Hz, 1H), 3.03-2.92 (m, 1H), 1.89 (s, 3H). $^{13}$C NMR (100 MHz, $CDCl_3$): δ = 172.7,

172.5, 169.3, 135.2, 134.3, 132.5, 130.5, 128.8, 128.6, 128.5, 128.4, 125.7 (q, $J$ = 280.9 Hz), 67.1, 54.5 (q, $J$ = 32.5 Hz), 31.5, 26.9. $^{19}F\{^1H\}$ NMR (376 MHz, $CDCl_3$): δ = -71.3 (s). HRMS (ESI) m/z: $[M+H]^+$ calcd for $C_{20}H_{18}ClF_3NO_4^+$ 428.0871, found 428.0865.

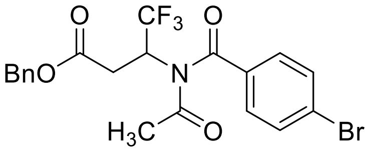


Compound **4j**: 37.3 mg, 79% yield, colorless oil. $^1H$ NMR (400 MHz, $CDCl_3$): δ = 7.65-7.58 (m, 2H), 7.57-7.50 (m, 2H), 7.42-7.30 (m, 5H), 5.67-5.51 (m, 1H), 5.23-5.09 (m, 2H), 3.62 (dd, $J$ = 11.48, 17.16 Hz, 1H), 3.03-2.93 (m, 1H), 1.90 (s, 3H). $^{13}C$ NMR (100 MHz, $CDCl_3$): δ = 172.7, 172.5, 169.3, 135.2, 134.3, 132.5, 130.5, 128.8, 128.6, 128.5, 128.4, 128.1 (q, $J$ = 240.2 Hz), 67.1, 54.2 (q, $J$ = 32.6 Hz), 31.5, 26.9. $^{19}F\{^1H\}$ NMR (376 MHz, $CDCl_3$): δ = -71.2 (s). HRMS (ESI) m/z: $[M+H]^+$ calcd for $C_{20}H_{18}BrF_3NO_4^+$ 472.0366, found 472.0361.

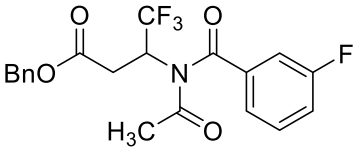


Compound **4k**: 35.3 mg, 86% yield, colorless oil. $^1H$ NMR (400 MHz, $CDCl_3$): δ = 7.48-7.40 (m, 3H), 7.39-7.30 (m, 6H), 5.66-5.51 (m, 1H), 5.22-5.11 (m, 2H), 3.64 (dd, $J$ = 11.32, 17.36 Hz, 1H), 3.02 (dd, $J$ = 4.24, 17.32 Hz, 1H), 1.90 (s, 3H). $^{13}C$ NMR (100 MHz, $CDCl_3$): δ = 172.8, 172.2 (d, $J$ = 2.7 Hz), 169.3, 164.0 (d, $J$ = 248.4 Hz), 137.7 (d, $J$ = 6.8 Hz), 135.2, 130.9 (d, $J$ = 7.9 Hz), 128.6, 128.5, 128.4, 125.7 (q, $J$ = 281.1 Hz), 124.7 (d, $J$ = 3.0 Hz), 120.7 (d, $J$ = 21.2 Hz), 116.2 (d, $J$ = 23.0 Hz), 67.1, 54.6 (q, $J$ = 33.0 Hz), 31.5, 26.9. $^{19}F\{^1H\}$ NMR (376 MHz, $CDCl_3$): δ = -71.2 (s), -110.2 (s). HRMS (ESI) m/z: $[M+H]^+$ calcd for $C_{20}H_{18}F_4NO_4^+$ 412.1166, found 412.1166.

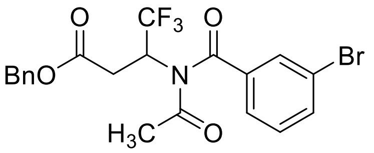


Compound **4l**: 36.8 mg, 78% yield, colorless oil. $^{1}$H NMR (400 MHz, $CDCl_3$): δ = 7.88-7.85 (m, 1H), 7.77-7.70 (m, 1H), 7.58-7.53 (m, 1H), 7.40-7.30 (m, 6H), 5.65-5.50 (m, 1H), 5.22-5.11 (m, 2H), 3.64 (dd, *J* = 11.32, 17.36 Hz, 1H), 3.01 (dd, *J* = 4.24, 17.32 Hz, 1H), 1.91 (s, 3H). $^{13}$C NMR (100 MHz, $CDCl_3$): δ = 172.8, 172.0, 169.3, 137.4, 136.4, 135.2, 132.0, 130.6, 128.6, 128.5, 128.4, 127.4, 125.7 (q, *J* = 280.8 Hz), 123.4, 67.1, 54.3 (q, *J* = 30.5 Hz), 31.5, 27.0. $^{19}$F{$^{1}$H} NMR (376 MHz, $CDCl_3$): δ = -71.1 (s). HRMS (ESI) m/z: $[M+H]^+$ calcd for $C_{20}H_{18}BrF_3NO_4^+$ 472.0366, found 472.0361.

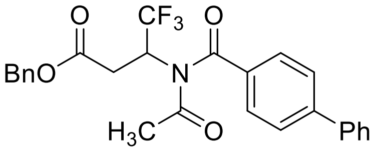


Compound **4m**: 18.3 mg, 39% yield, colorless oil. $^{1}$H NMR (400 MHz, $CDCl_3$): δ = 7.80-7.74 (m, 2H), 7.73-7.67 (m, 2H), 7.66-7.61 (m, 2H), 7.55-7.49 (m, 2H), 7.48-7.42 (m, 1H), 7.41-7.31 (m, 5H), 5.77-5.63 (m, 1H), 5.23-5.11 (m, 2H), 3.68-3.55 (m, 1H), 3.06-2.95 (m, 1H), 1.92 (s, 3H). $^{13}$C NMR (100 MHz, $CDCl_3$): δ = 173.2, 172.9, 169.3, 146.5, 139.3, 135.3, 134.1, 129.9, 129.1, 128.7, 128.6, 128.5, 128.4, 127.7, 127.3, 125.8 (q, *J* = 280.5 Hz), 67.1, 54.0 (q, *J* = 31.9 Hz), 31.7, 27.0. $^{19}$F{$^{1}$H} NMR (376 MHz, $CDCl_3$): δ = -71.2 (s). HRMS (ESI) m/z: $[M+H]^+$ calcd for $C_{26}H_{23}F_3NO_4^+$ 470.1574, found 470.1580.

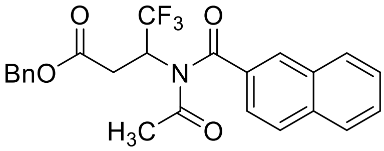


Compound **4n**: 34.6 mg, 78% yield, colorless oil. $^{1}$H NMR (400 MHz, $CDCl_3$): δ = 8.23 (s, 1H), 7.99-7.87 (m, 3H), 7.80-7.72 (m, 1 H), 7.71-7.57 (m, 2H), 7.39-7.30 (m, 5H), 5.83-5.67 (m, 1H),

5.23-5.12 (m, 2H), 3.68 (dd, *J* = 11.32, 17.08 Hz, 1H), 3.09-2.97 (m, 1H), 1.87 (s, 3H). $^{13}$C NMR (100 MHz, $CDCl_3$): δ = 173.5, 173.0, 135.6, 135.3, 132.8, 132.4, 130.9, 129.5, 129.3, 129.1, 128.6, 128.4, 128.3, 127.9, 127.4, 125.9 (q, *J* = 281.2 Hz), 124.6, 67.0, 54.3 (q, *J* = 31.8 Hz), 31.7, 27.0. $^{19}$F{$^1$H} NMR (376 MHz, $CDCl_3$): δ = -71.2 (s). HRMS (ESI) m/z: [M+H]$^+$ calcd for $C_{24}H_{21}F_3NO_4^+$ 444.1417, found 444.1414.

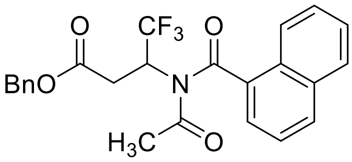


Compound **4o**: 23.9 mg, 54% yield, colorless oil. $^1$H NMR (400 MHz, $CDCl_3$): δ = 8.34-8.26 (m, 1H), 8.07-8.00 (m, 1H), 7.97-7.90 (m, 1H), 7.71-7.53 (m, 3H), 7.48-7.41 (m, 1H), 7.40-7.31 (m, 5H), 5.88-5.73 (m, 1H), 5.21 (s, 2H), 3.91-3.58 (m, 1H), 3.13-3.02 (m, 1H), 1.72 (s, 3H). $^{13}$C NMR (100 MHz, $CDCl_3$): δ = 173.6, 172.6, 169.5, 135.3, 133.9, 133.3, 133.2, 130.4, 128.8, 128.7, 128.6, 128.5, 128.4, 127.8, 127.2, 125.9 (q, *J* = 281.5 Hz), 124.9, 124.7, 67.1, 53.7 (q, *J* = 31.9 Hz), 29.7, 27.2. $^{19}$F{$^1$H} NMR (376 MHz, $CDCl_3$): δ = -70.7 (s). HRMS (ESI) m/z: [M+H]$^+$ calcd for $C_{24}H_{21}F_3NO_4^+$ 444.1417, found 444.1411.

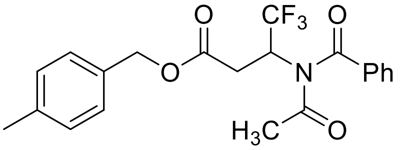


Compound **4q**: 28.5 mg, 70% yield, colorless oil. $^1$H NMR (400 MHz, $CDCl_3$): δ = 7.72-7.66 (m, 2H), 7.65-7.59 (m, 1H), 7.51-7.44 (m, 2H), 7.27-7.20 (m, 2H), 7.19-7.13 (m, 2H), 5.73-5.58 (m, 1H), 5.17-5.05 (m, 2H), 3.62 (dd, *J* = 11.20, 17.24 Hz, 1H), 3.00 (dd, *J* = 4.32, 17.24 Hz, 1H), 2.36 (s, 3H), 1.85 (s, 3H). $^{13}$C NMR (100 MHz, $CDCl_3$): δ = 173.4, 173.0, 169.3, 138.4, 135.7, 133.5, 132.3, 129.3, 129.1, 128.6, 125.8 (q, *J* = 281.0 Hz), 67.0, 54.0 (q, *J* = 32.0 Hz), 31.6, 27.0, 21.2. $^{19}$F{$^1$H} NMR (376 MHz, $CDCl_3$): δ = -71.1 (s). HRMS (ESI) m/z: [M+H]$^+$ calcd for $C_{21}H_{21}F_3NO_4^+$

408.1417, found 408.1421.

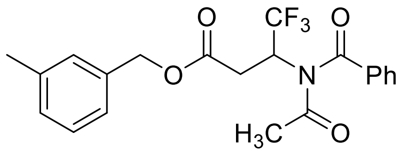


Compound **4r**: 24.8 mg, 61% yield, colorless oil. $^{1}$H NMR (400 MHz, $CDCl_3$): δ = 7.73-7.66 (m, 2H), 7.65-7.58 (m, 1H), 7.52-7.44 (m, 2H), 7.28-7.22 (m, 1H), 7.21-7.10 (m, 3H), 5.75-5.58 (m, 1H), 5.18-5.06 (m, 2H), 3.64 (dd, $J$ = 11.24, 17.24 Hz, 1H), 3.02 (dd, $J$ = 4.32, 17.28 Hz, 1H), 2.36 (s, 3H), 1.85 (s, 3H). $^{13}$C NMR (100 MHz, $CDCl_3$): δ = 173.4, 173.0, 169.3, 138.3, 135.6, 135.2, 133.6, 129.3, 129.2, 129.1 128.5, 125.8 (q, $J$ = 281.4 Hz), 125.5, 67.1, 54.0 (q, $J$ = 32.9 Hz), 31.6, 27.0, 21.3. $^{19}$F{$^{1}$H} NMR (376 MHz, $CDCl_3$): δ = -71.2 (s). HRMS (ESI) m/z: $[M+H]^+$ calcd for $C_{21}H_{21}F_3NO_4^+$ 408.1417, found 408.1418.

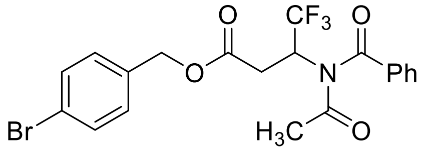


Compound **4s**: 33.5 mg, 71% yield, colorless oil. $^{1}$H NMR (400 MHz, $CDCl_3$): δ = 7.70-7.60 (m, 3H), 7.51-7.44 (m, 4H), 7.24-7.18 (m, 2H), 5.73-5.59 (m, 1H), 5.10 (s, 2H), 3.63 (dd, $J$ = 11.24, 17.28 Hz, 1H), 3.02 (dd, $J$ = 4.40, 17.28 Hz, 1H), 1.85 (s, 3H). $^{13}$C NMR (100 MHz, $CDCl_3$): δ = 173.4, 173.0, 169.2, 135.5, 134.3, 133.7, 131.7, 130.1, 129.2, 129.1, 125.8 (q, $J$ = 280.9 Hz), 122.5, 66.2, 53.9 (q, $J$ = 34.0 Hz), 31.6, 27.0. $^{19}$F{$^{1}$H} NMR (376 MHz, $CDCl_3$): δ = -71.2 (s). HRMS (ESI) m/z: $[M+H]^+$ calcd for $C_{20}H_{18}BrF_3NO_4^+$ 472.0366, found 472.0372.

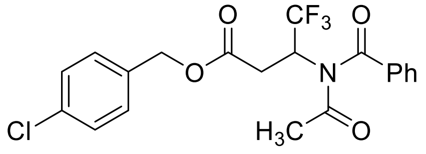


Compound **4t**: 19.7 mg, 46% yield, colorless oil. $^{1}$H NMR (400 MHz, $CDCl_3$): δ = 7.70-7.59 (m, 3H), 7.52-7.43 (m, 2H), 7.35-7.30 (m, 3H), 7.27-7.23 (m, 1H), 5.72-5.59 (m, 1H), 5.12 (s, 2H), 3.63

(dd, $J$ = 11.12, 17.20 Hz, 1H), 3.04-2.95 (m, 1H), 1.85 (s, 3H). $^{13}C$ NMR (100 MHz, $CDCl_3$): δ = 173.4, 173.0, 169.2, 135.5, 134.4, 133.8, 133.7, 129.8, 129.3, 129.2, 128.8, 125.8 (q, $J$ = 281.0 Hz), 66.2, 53.9 (q, $J$ = 32.1 Hz), 31.6, 27.0. $^{19}F\{^1H\}$ NMR (376 MHz, $CDCl_3$): δ = -71.2 (s). HRMS (ESI) m/z: $[M+H]^+$ calcd for $C_{20}H_{18}ClF_3NO_4^+$ 428.0871, found 428.0875.

## 5. $^{1}H$, $^{13}C$, $^{19}F$ NMR spectra for compounds 4

$^{1}H$ NMR (400 MHz, $CDCl_3$) of **4a**:

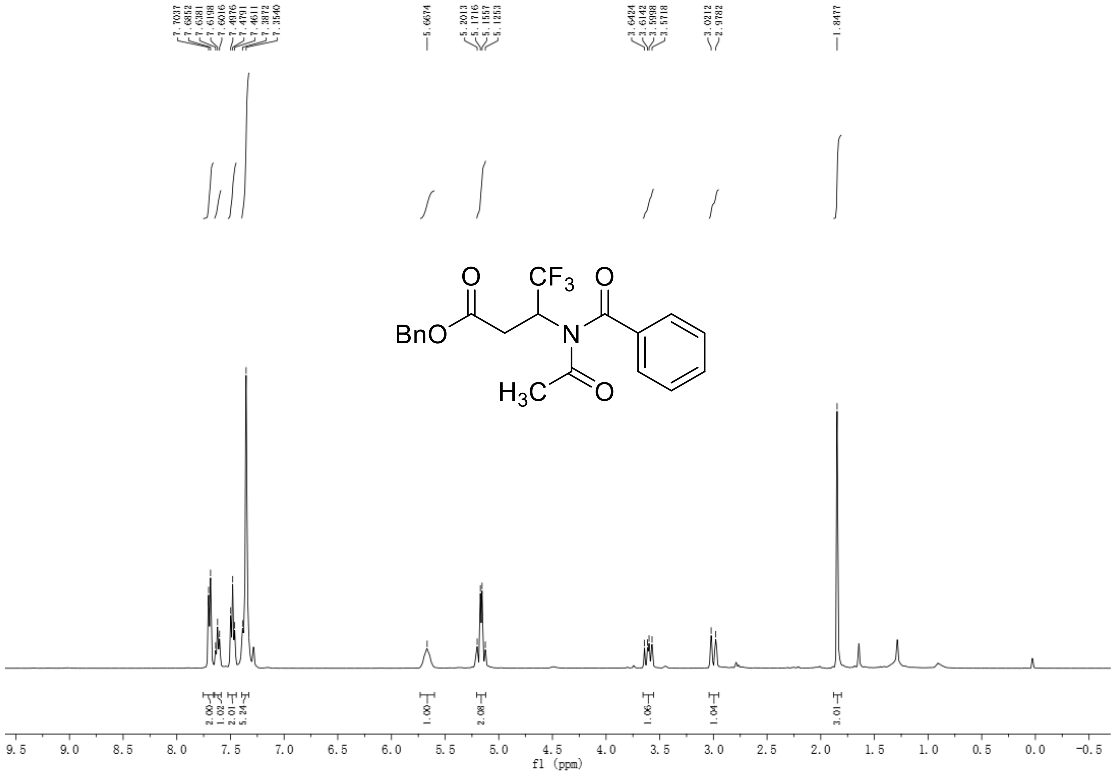

$^{13}C$ NMR (100 MHz, $CDCl_3$) of **4a**:

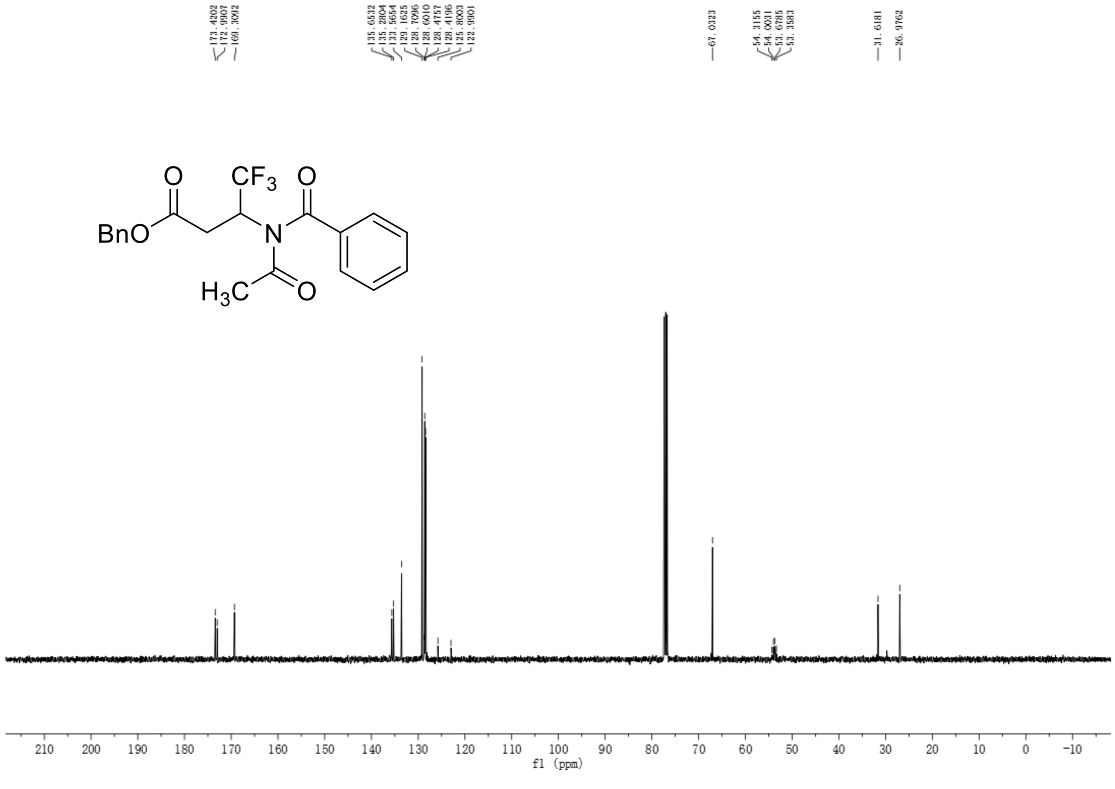

$^{19}F\{^{1}H\}$ NMR (376 MHz, $CDCl_3$) of **4a**:

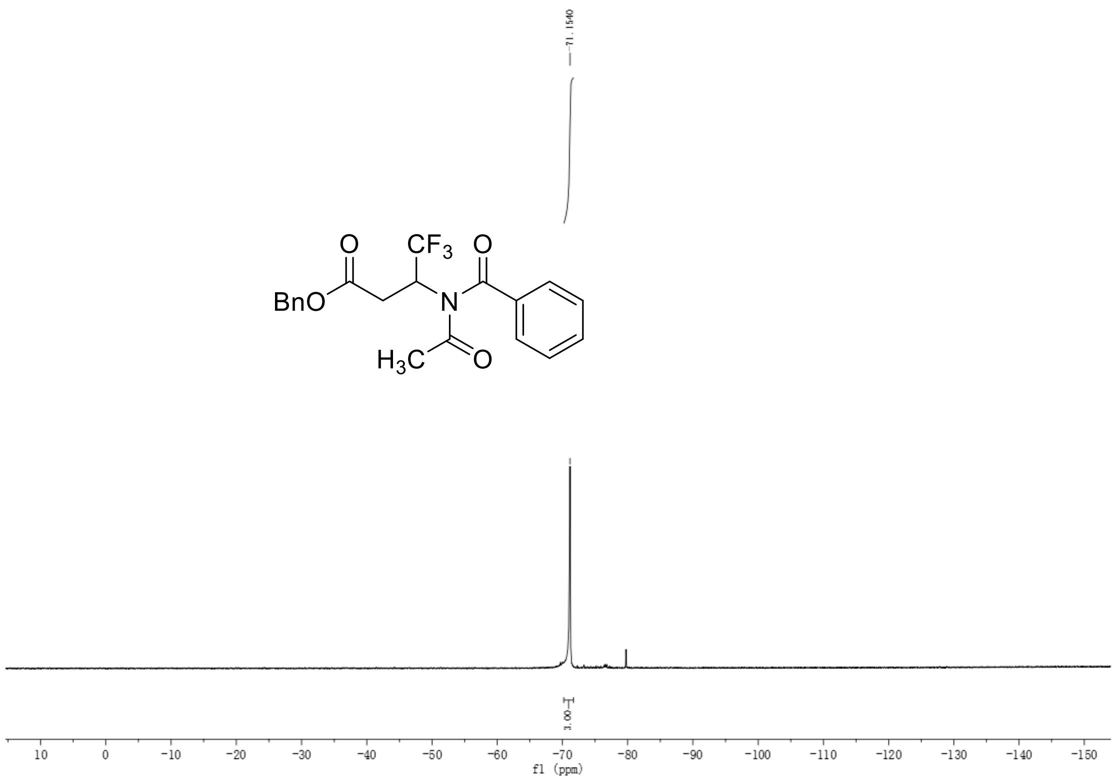


$^{1}H$ NMR (400 MHz, $CDCl_3$) of **4b**:

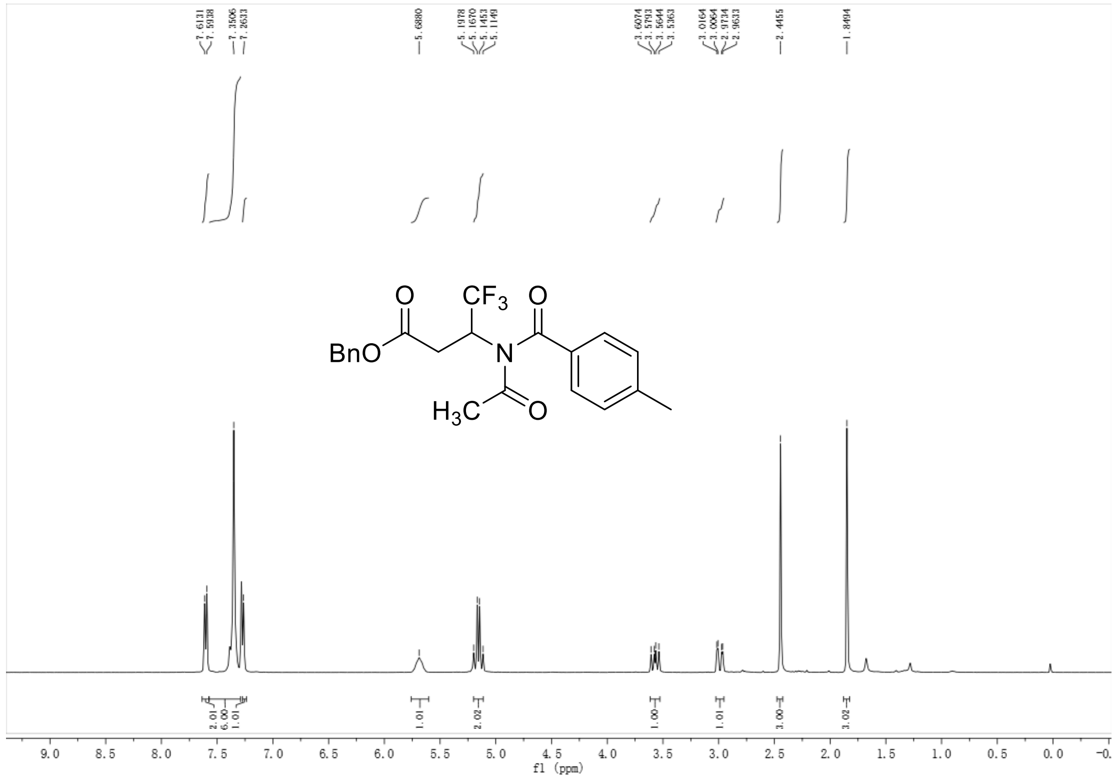

$^{13}$C NMR (100 MHz, $CDCl_3$) of **4b**:

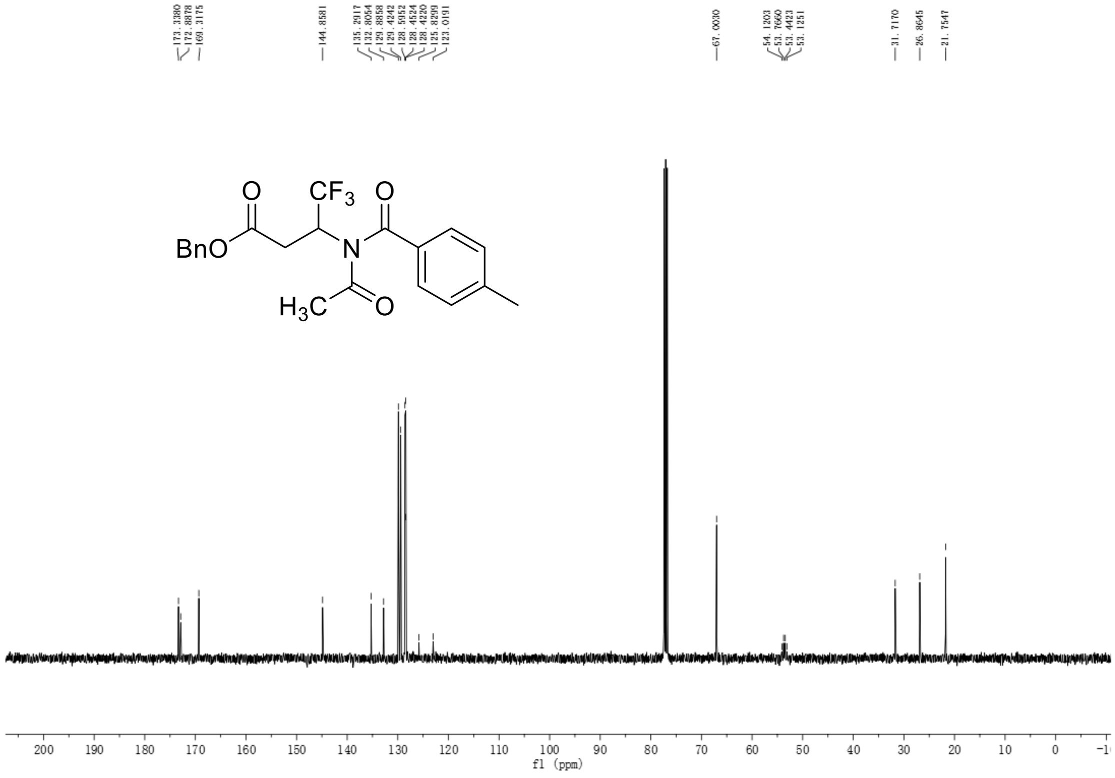


$^{19}$F{$^{1}$H} NMR (376 MHz, $CDCl_3$) of **4b**:

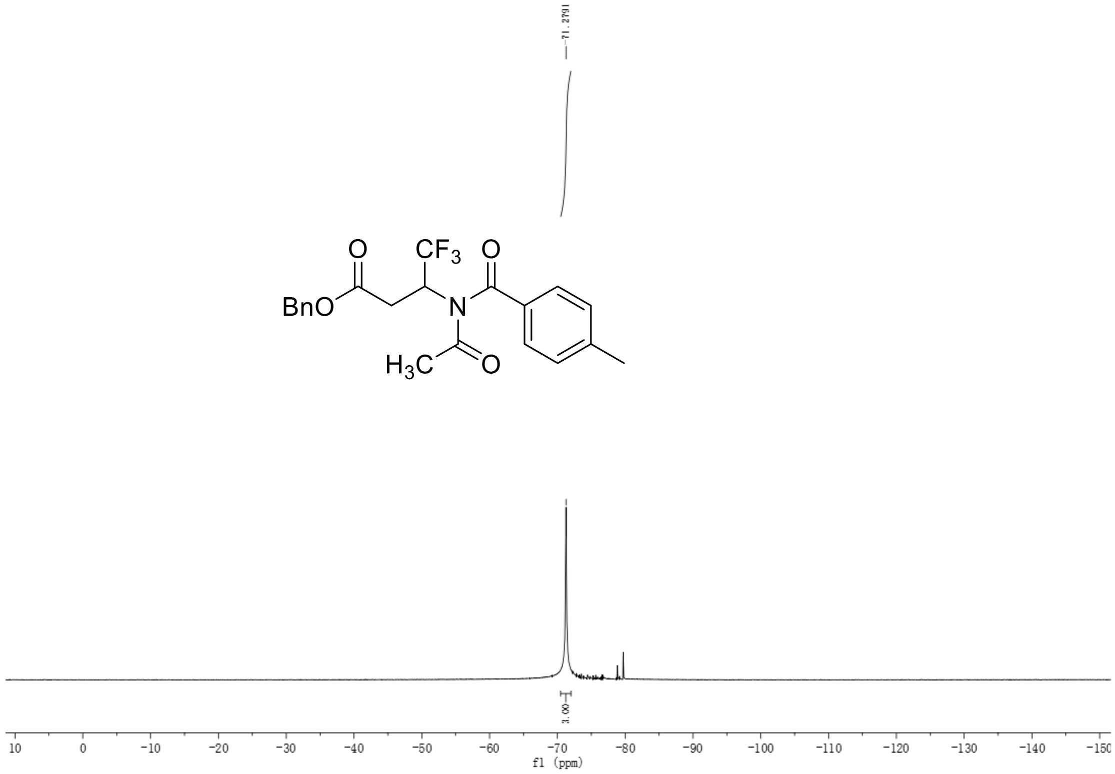

$^{1}$H NMR (400 MHz, $CDCl_3$) of **4c**:

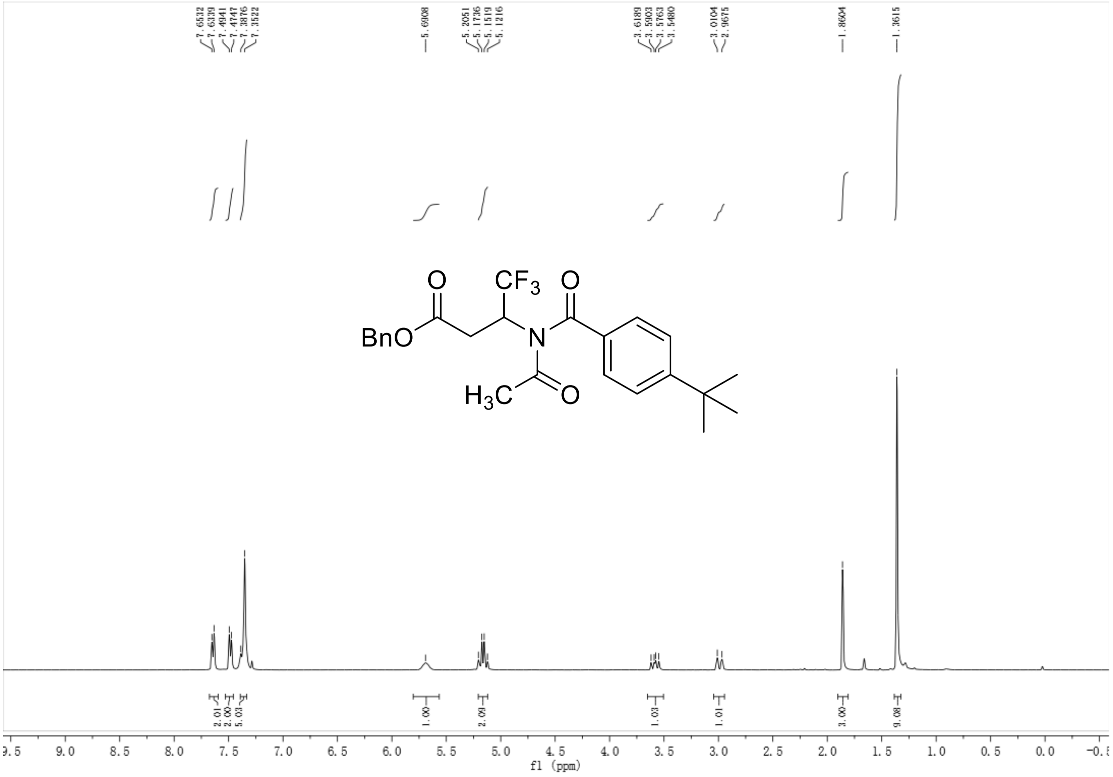

$^{13}$C NMR (100 MHz, $CDCl_3$) of **4c**:

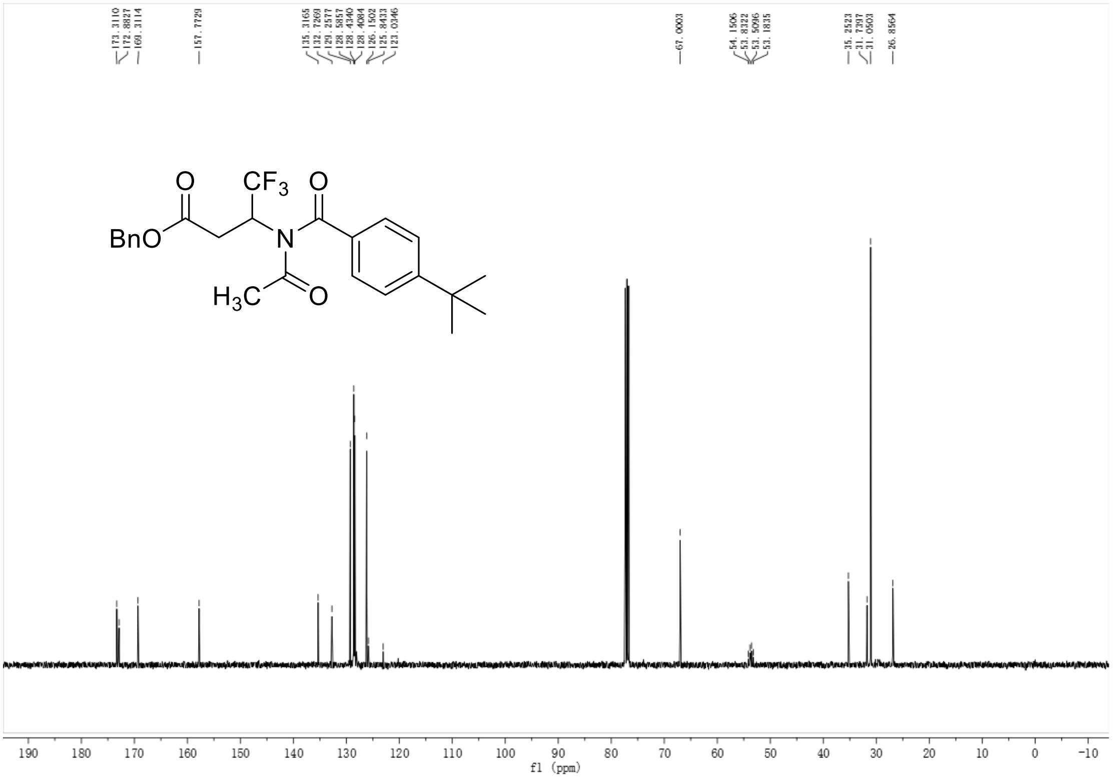

$^{19}F\{^1H\}$ NMR (376 MHz, $CDCl_3$) of **4c**:

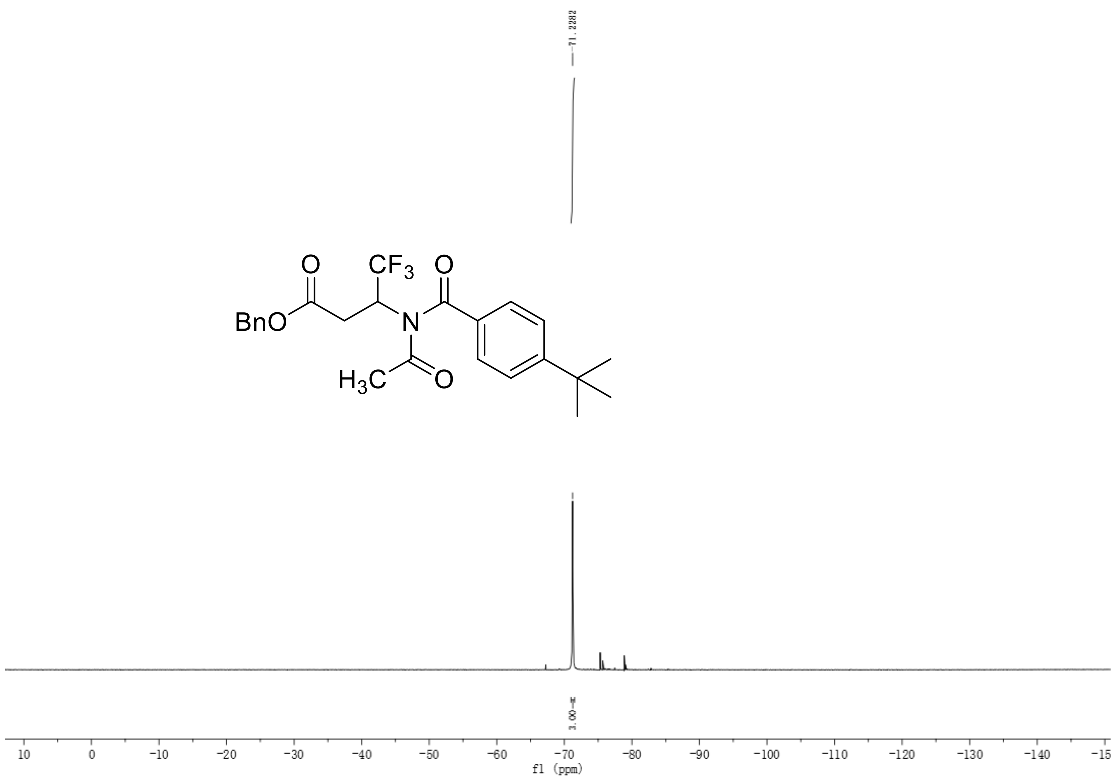

$^1H$ NMR (400 MHz, $CDCl_3$) of **4d**:

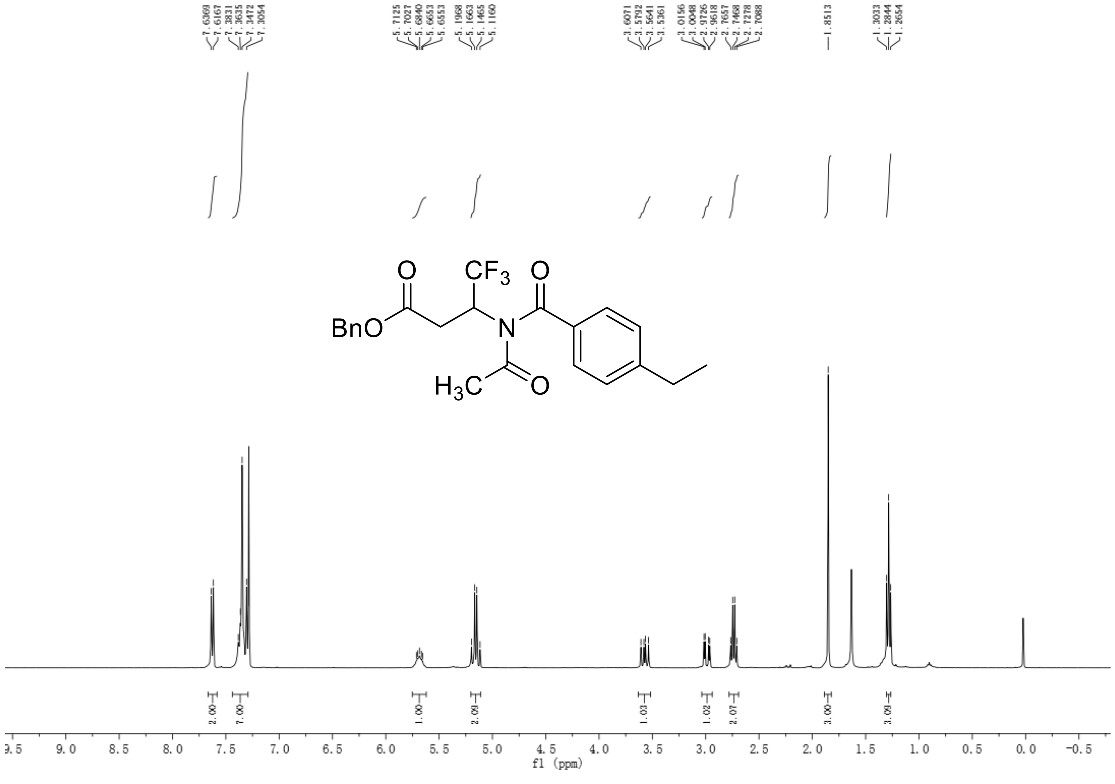

[13] C NMR (100 MHz, $CDCl_3$) of **4d**:

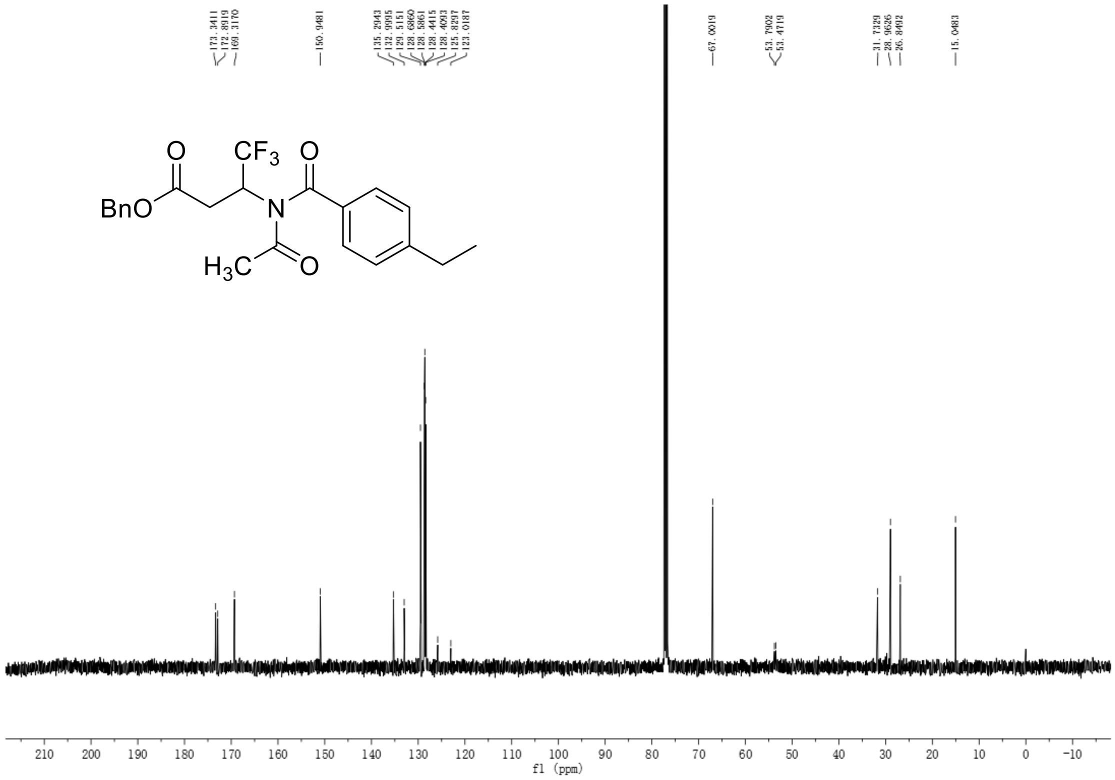


[19] F{[1] H} NMR (376 MHz, $CDCl_3$) of **4d**:

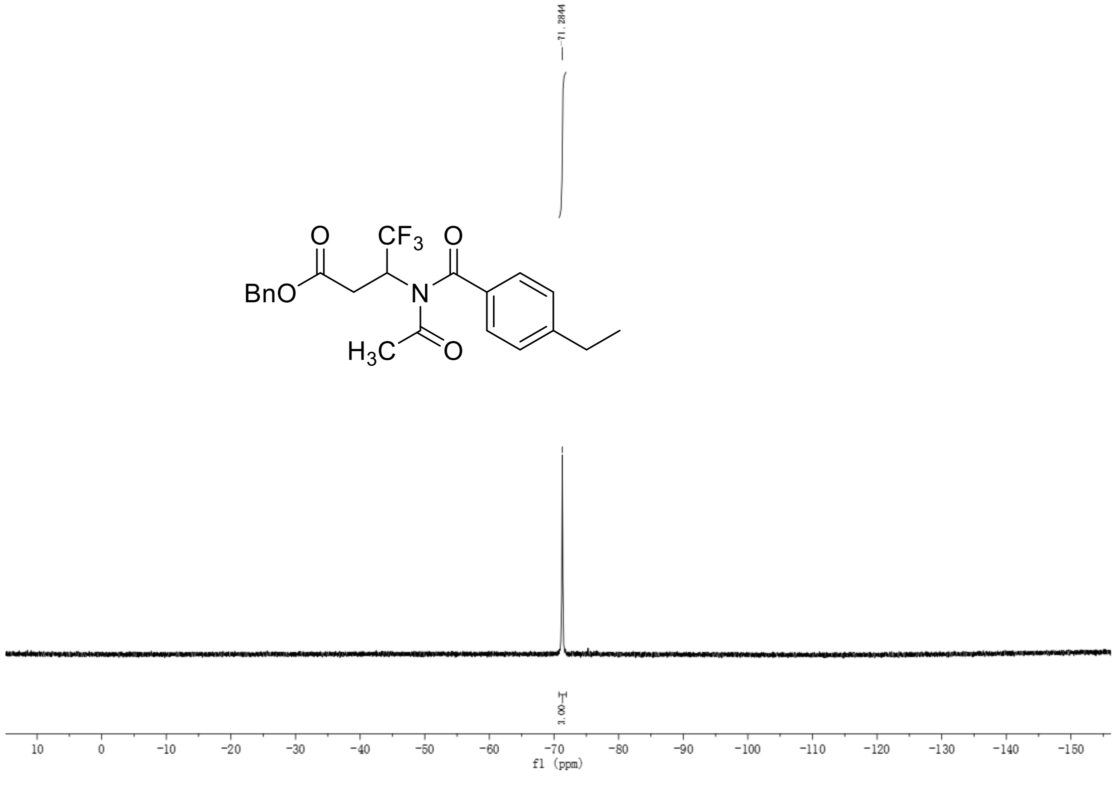

$^{1}$H NMR (400 MHz, $CDCl_3$) of **4e**:

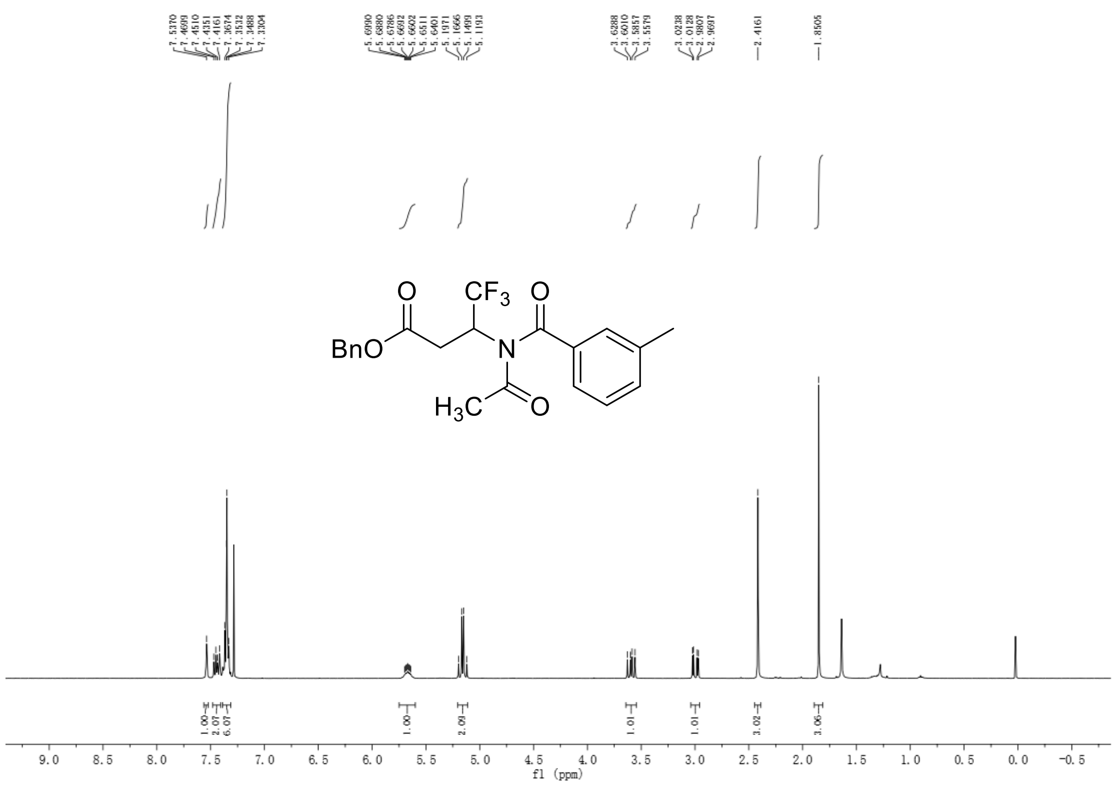

$^{13}$C NMR (100 MHz, $CDCl_3$) of **4e**:

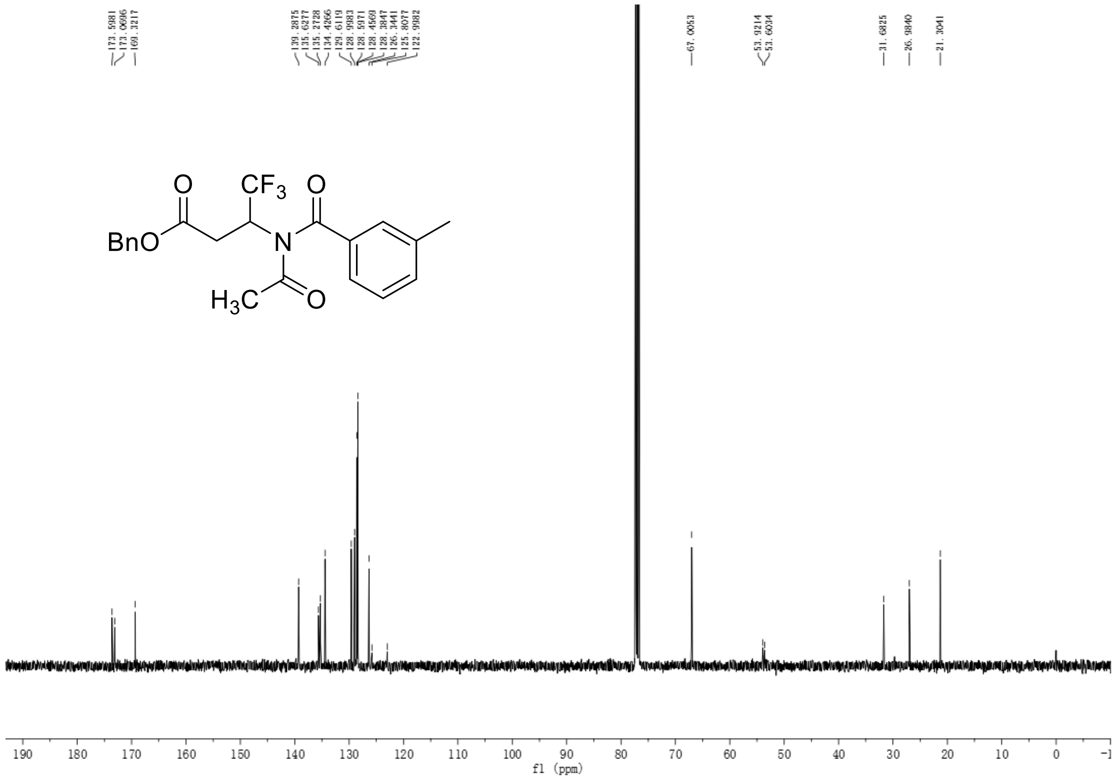

$^{19}F\{^1H\}$ NMR (376 MHz, $CDCl_3$) of **4e**:

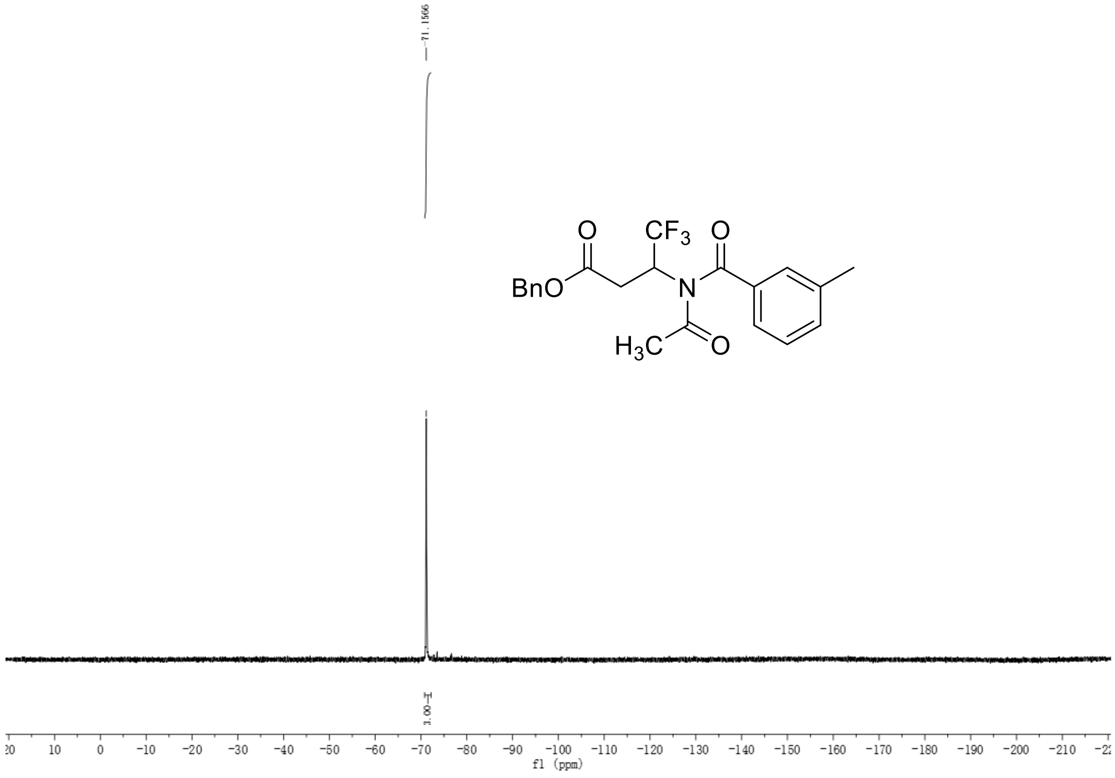


$^1H$ NMR (400 MHz, $CDCl_3$) of **4h**:

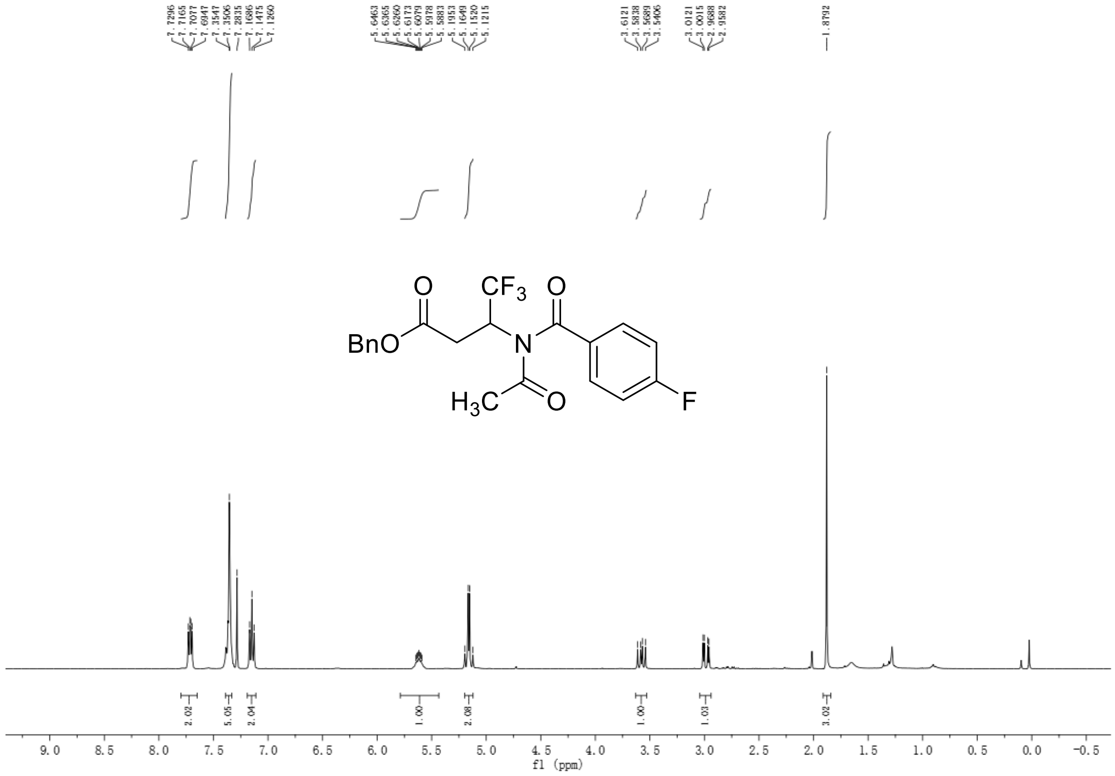

$^{13}C$ NMR (100 MHz, $CDCl_3$) of **4h**:

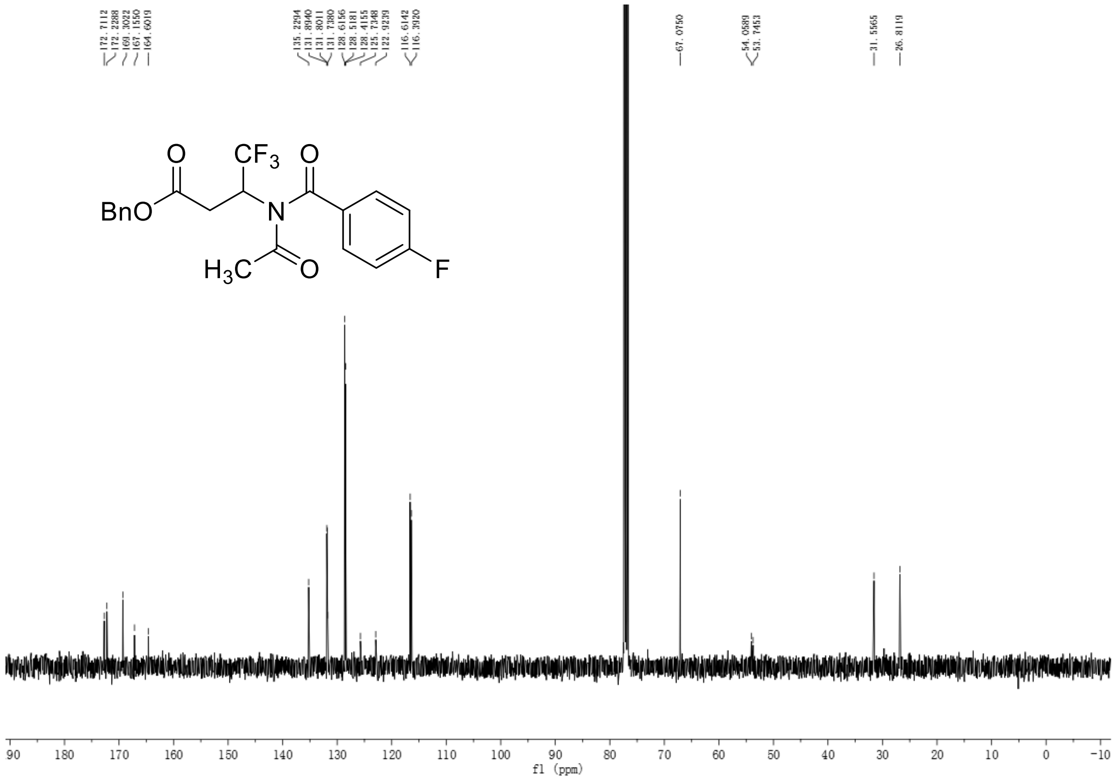


$^{19}F\{^1H\}$ NMR (376 MHz, $CDCl_3$) of **4h**:

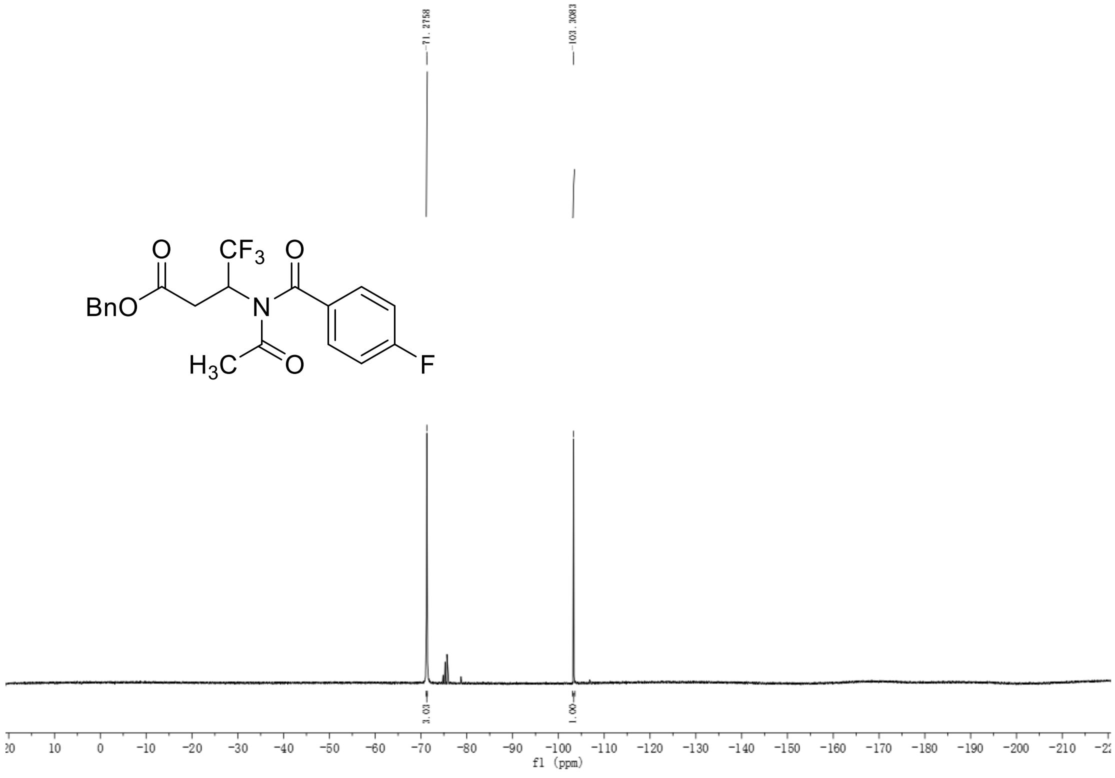

$^{1}H$ NMR (400 MHz, $CDCl_3$) of **4i**:

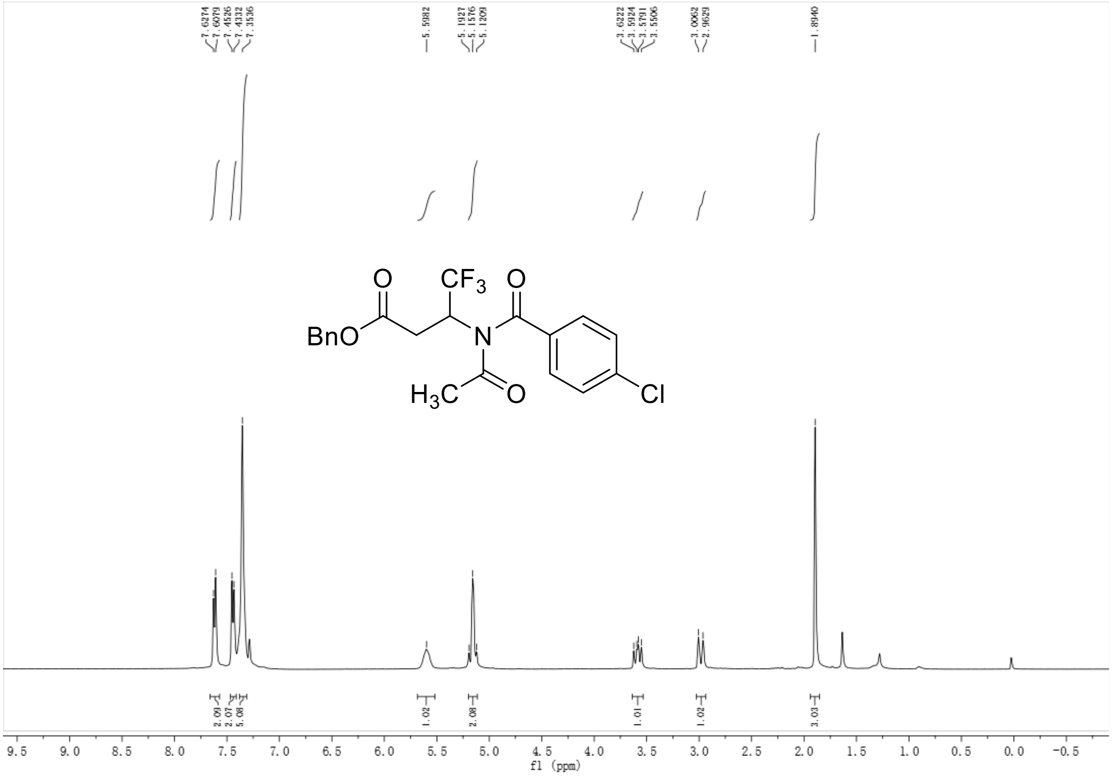

$^{13}C$ NMR (100 MHz, $CDCl_3$) of **4i**:

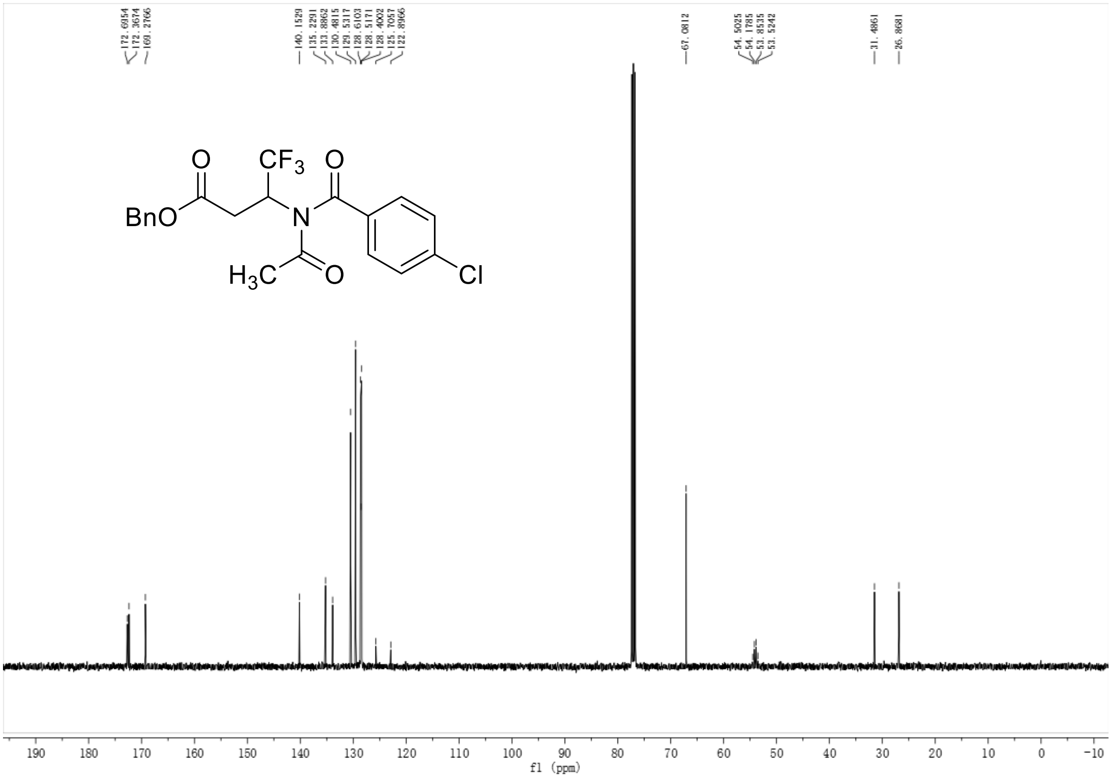

$^{19}F\{^1H\}$ NMR (376 MHz, $CDCl_3$) of **4i**:

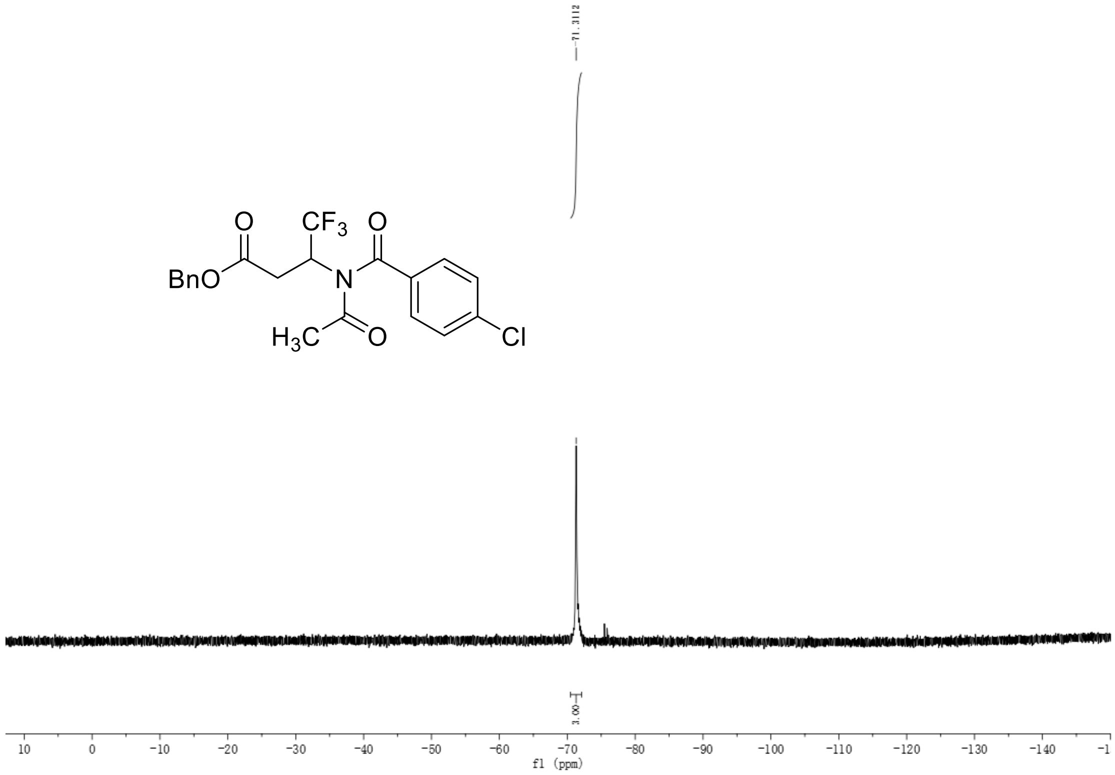


$^1H$ NMR (400 MHz, $CDCl_3$) of **4j**:

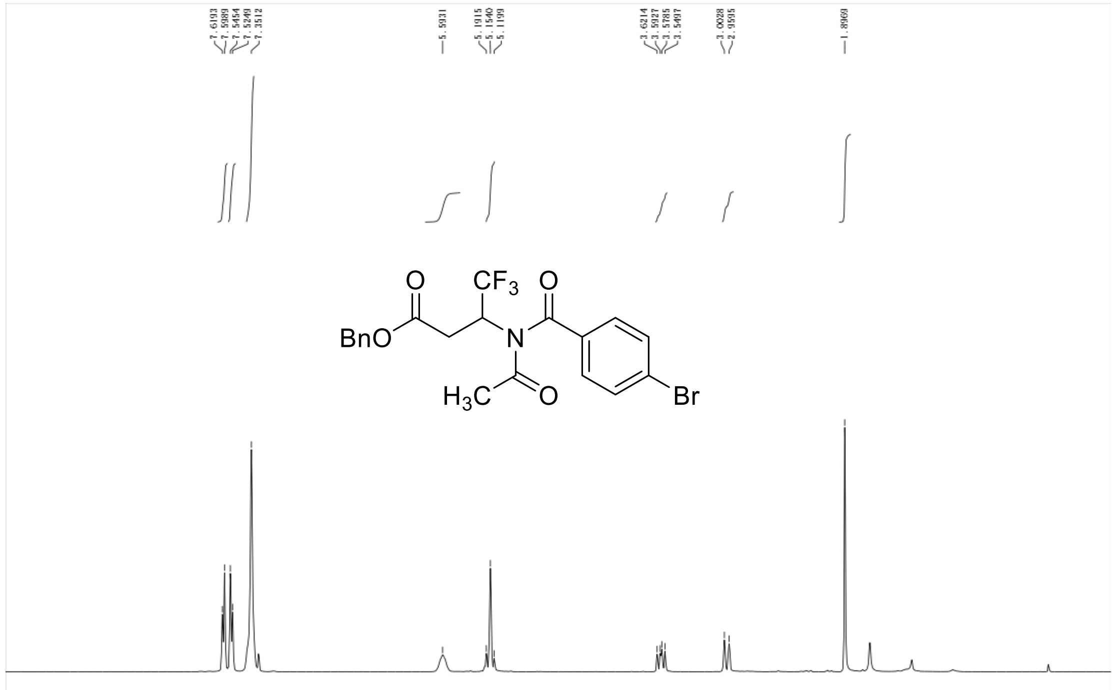

$^{13}$C NMR (100 MHz, $CDCl_3$) of **4j**:

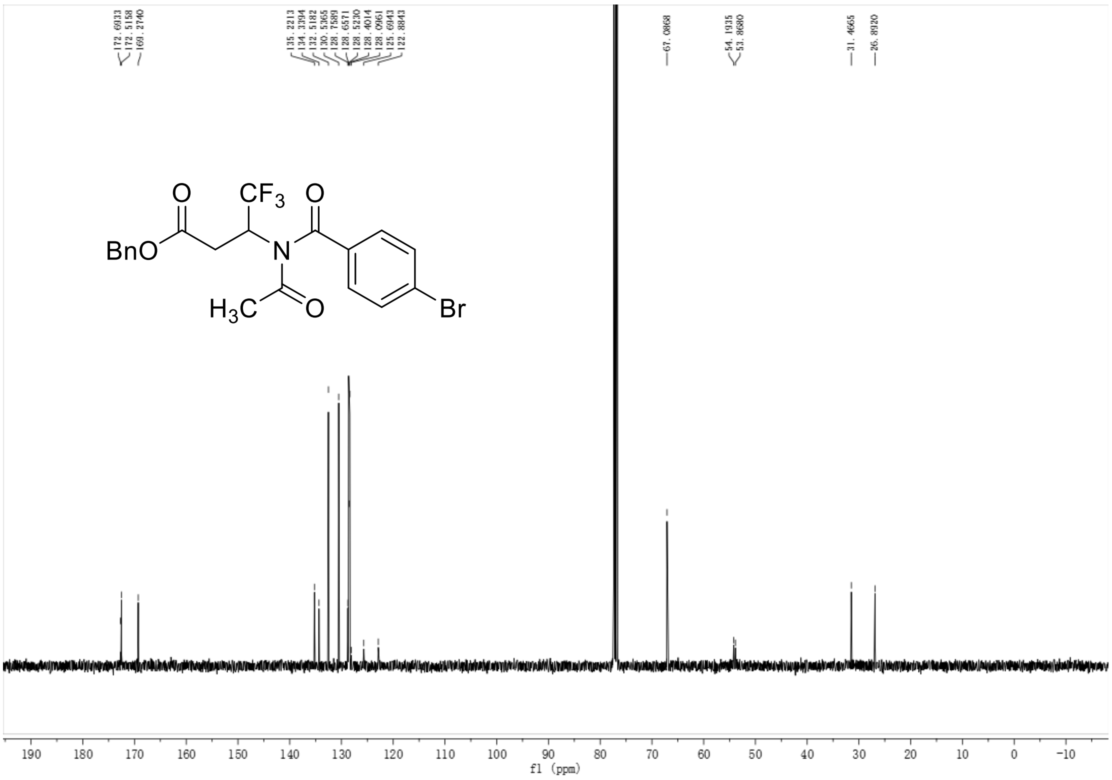

$^{19}$F{$^{1}$H} NMR (376 MHz, $CDCl_3$) of **4j**:

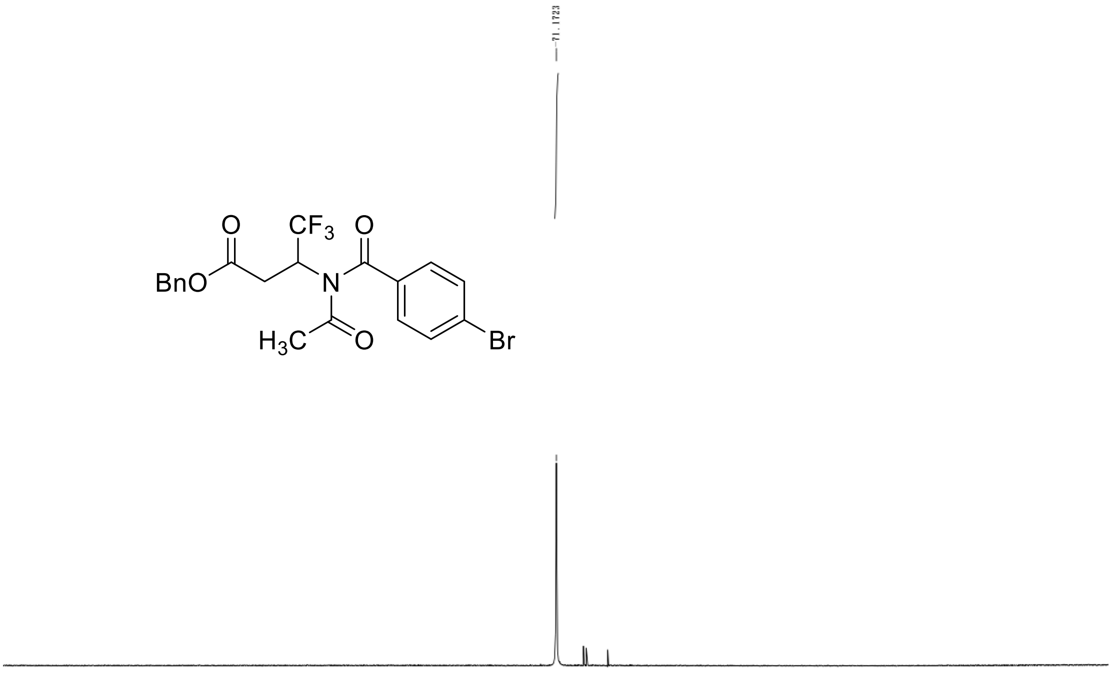

$^{1}$H NMR (400 MHz, $CDCl_3$) of **4k**:

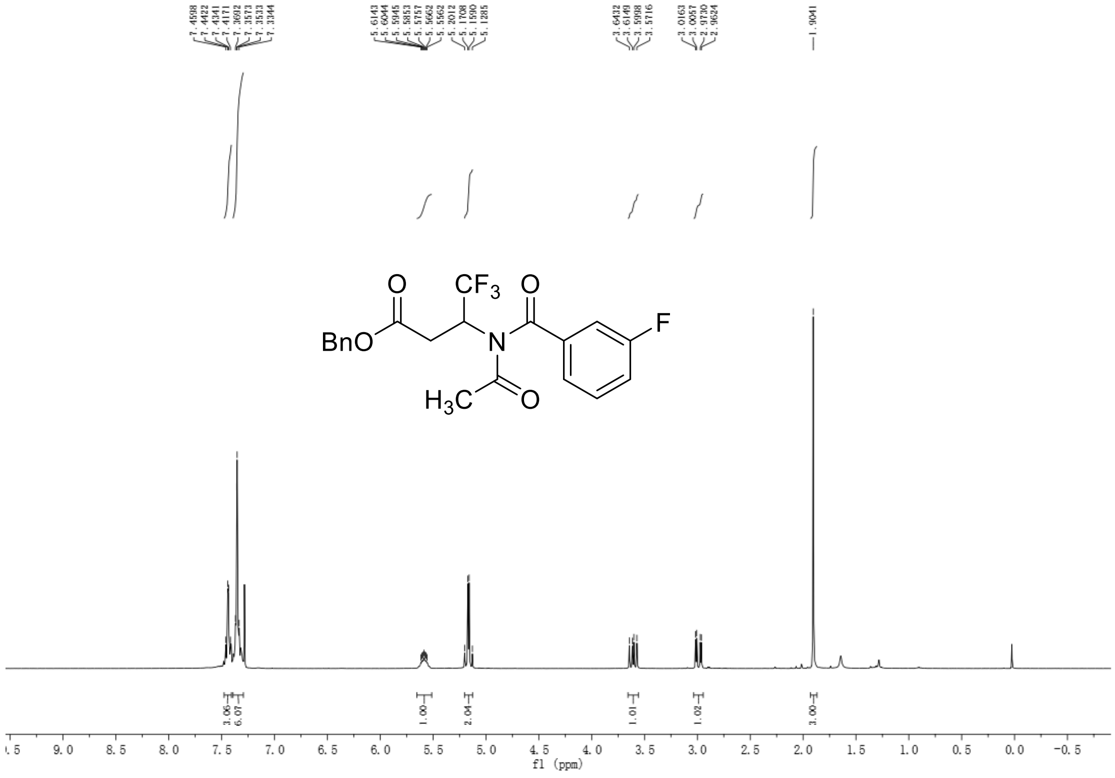

$^{13}$C NMR (100 MHz, $CDCl_3$) of **4k**:

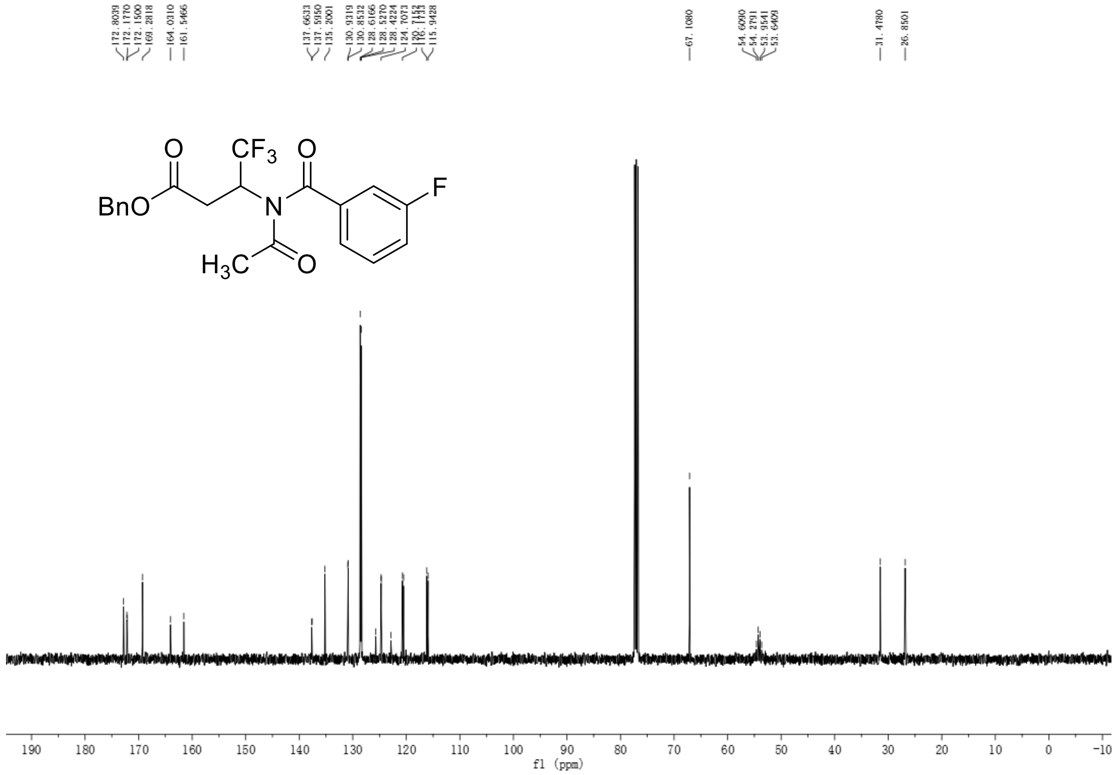

$^{19}F\{^1H\}$ NMR (376 MHz, $CDCl_3$) of **4k**:

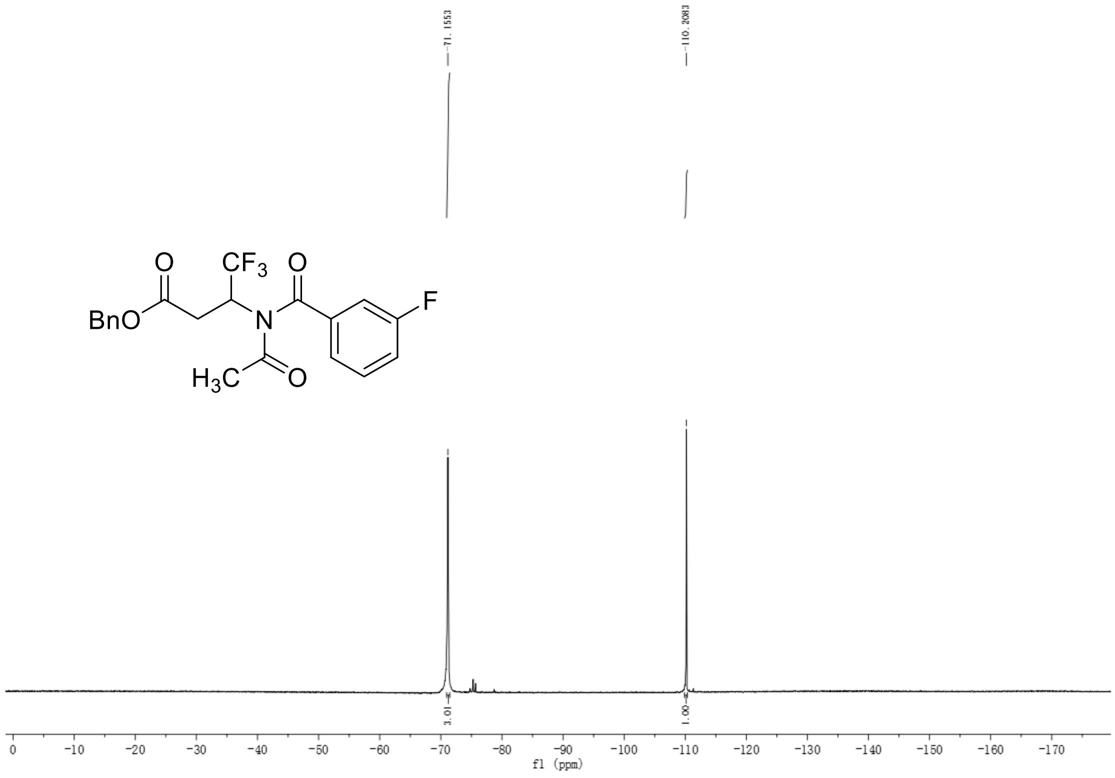


$^1H$ NMR (400 MHz, $CDCl_3$) of **4l**:

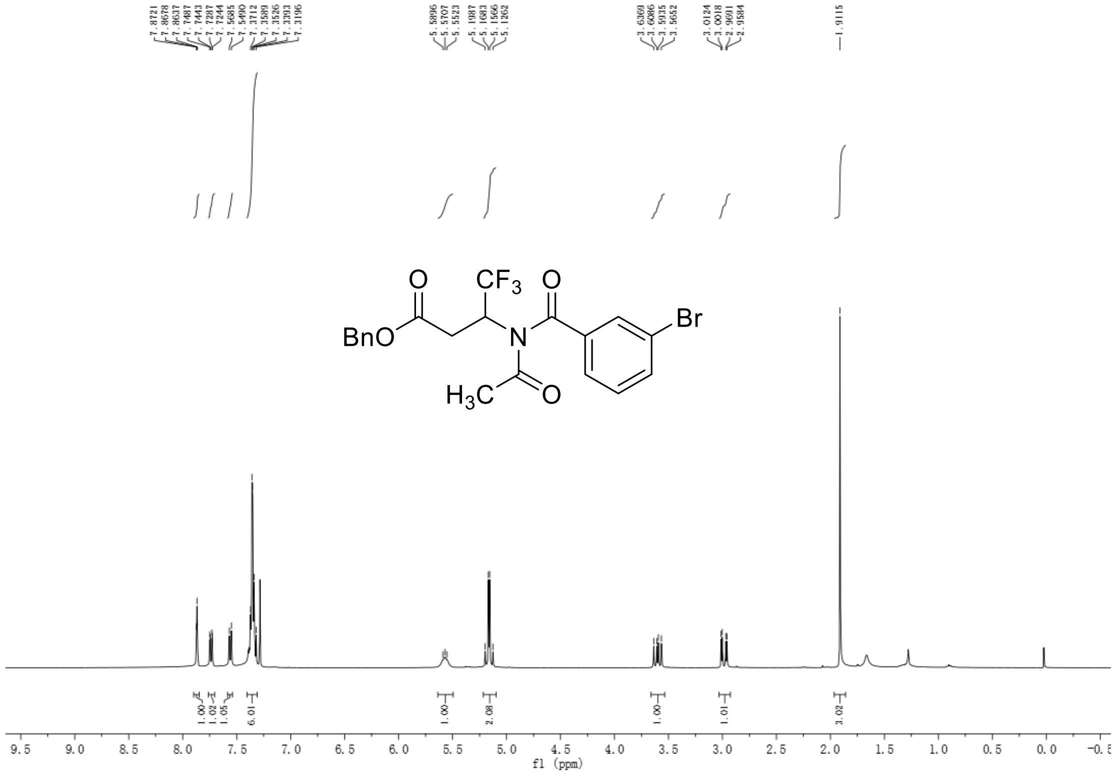

$^{13}C$ NMR (100 MHz, $CDCl_3$) of **4l**:

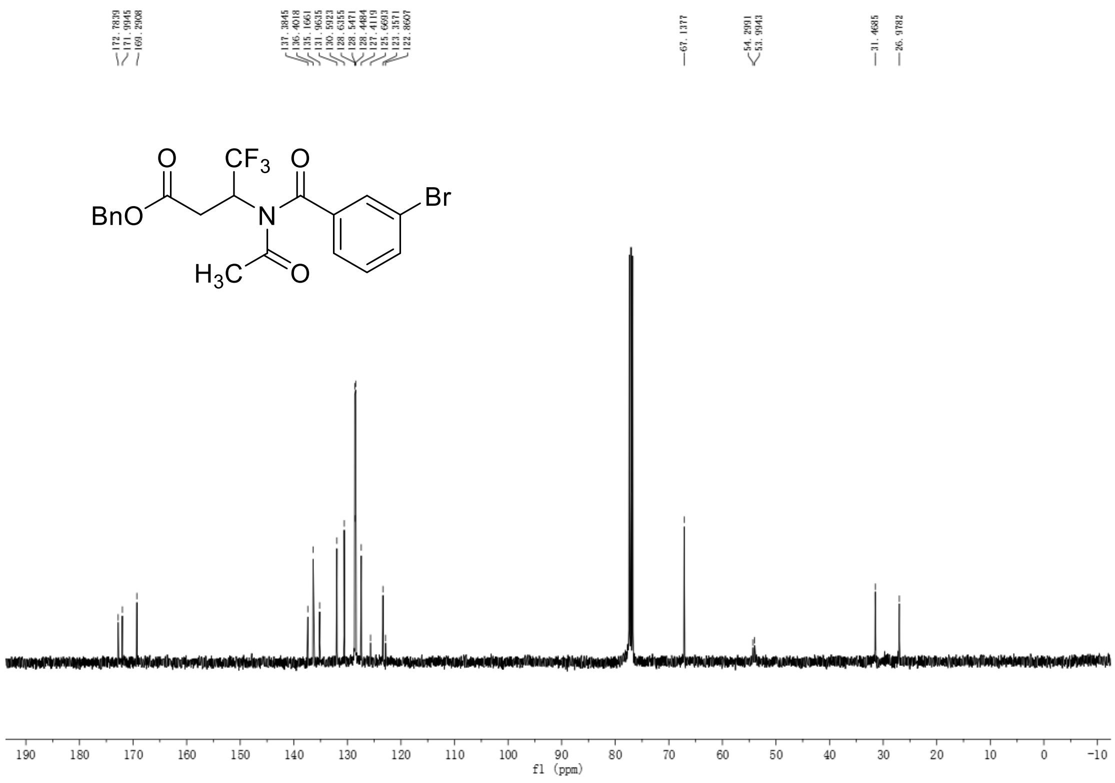


$^{19}F\{^{1}H\}$ NMR (376 MHz, $CDCl_3$) of **4l**:

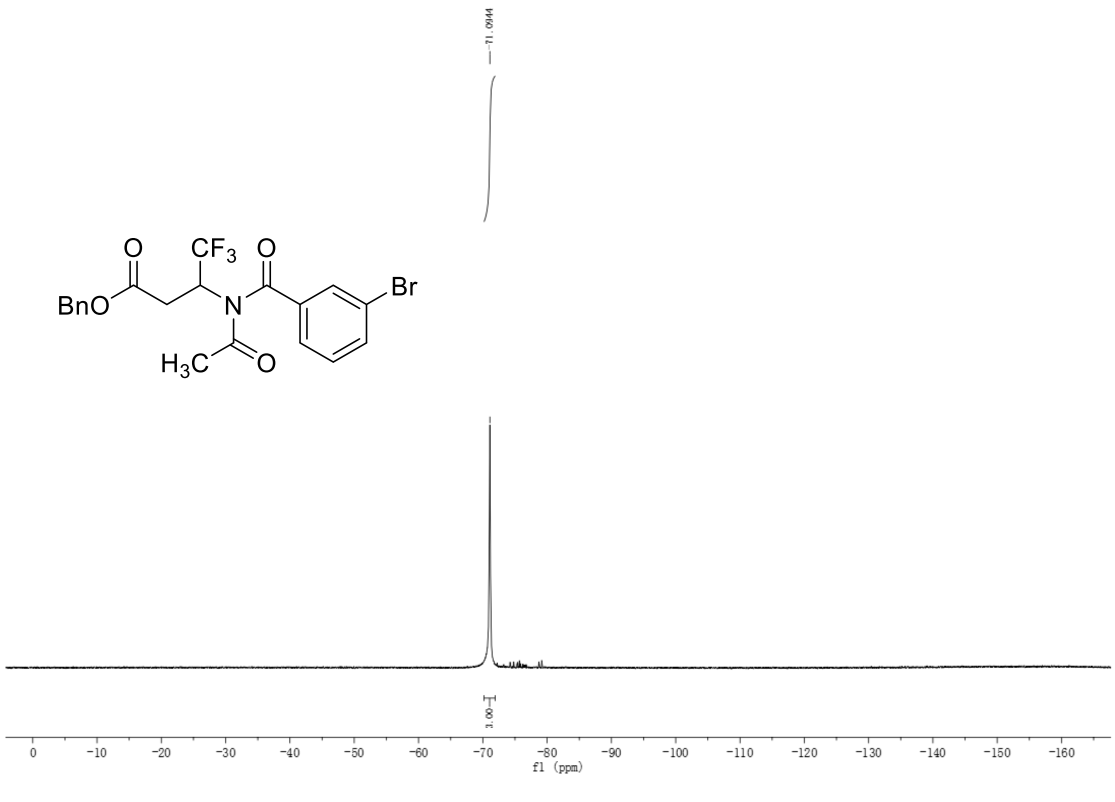

$^{1}$H NMR (400 MHz, $CDCl_3$) of **4m**:

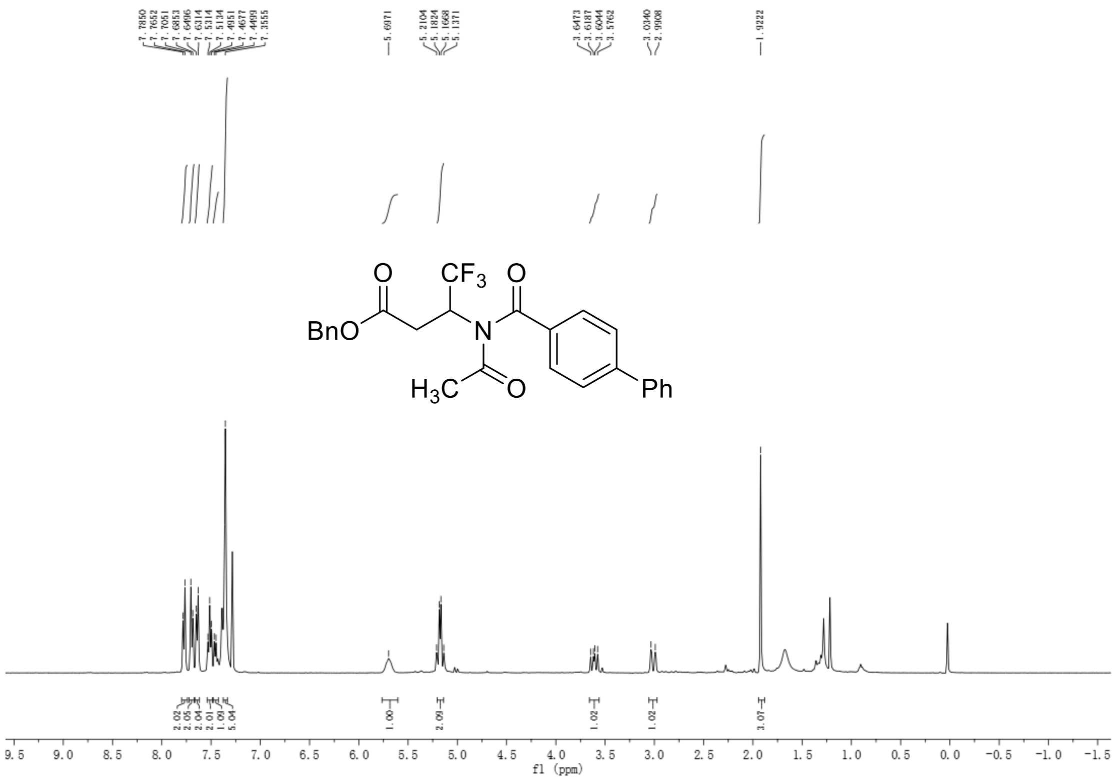

$^{13}$C NMR (100 MHz, $CDCl_3$) of **4m**:

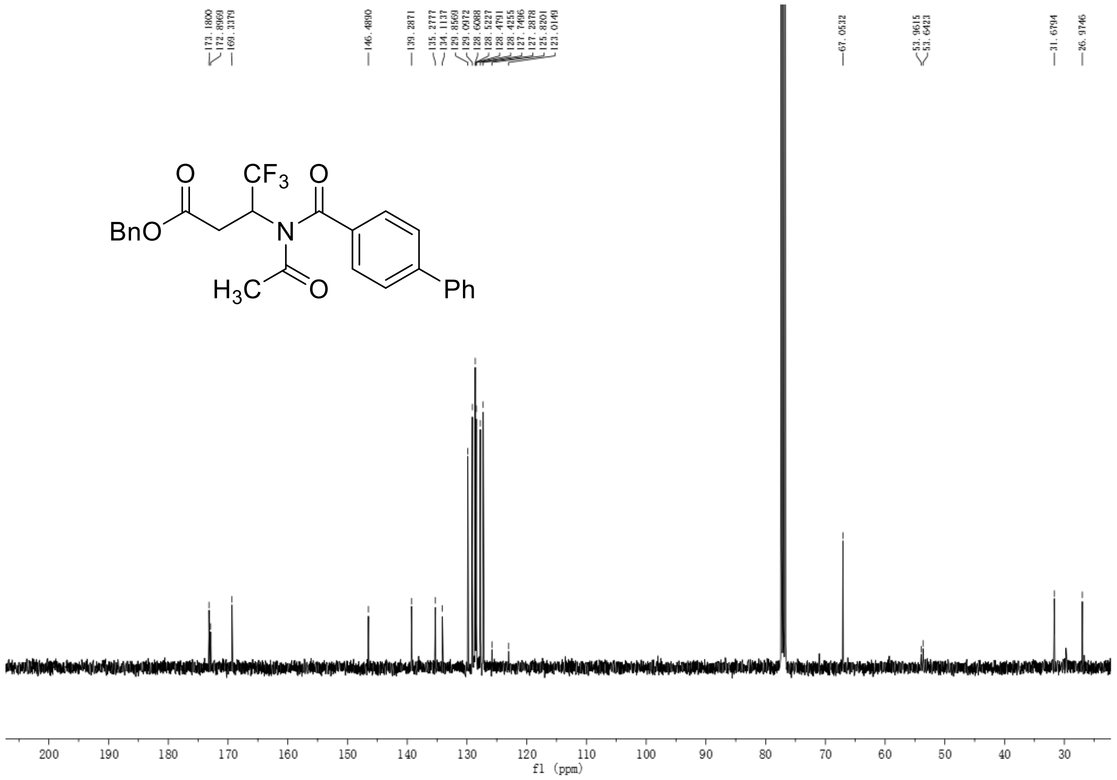

$^{19}F\{^1H\}$ NMR (376 MHz, $CDCl_3$) of **4m**:

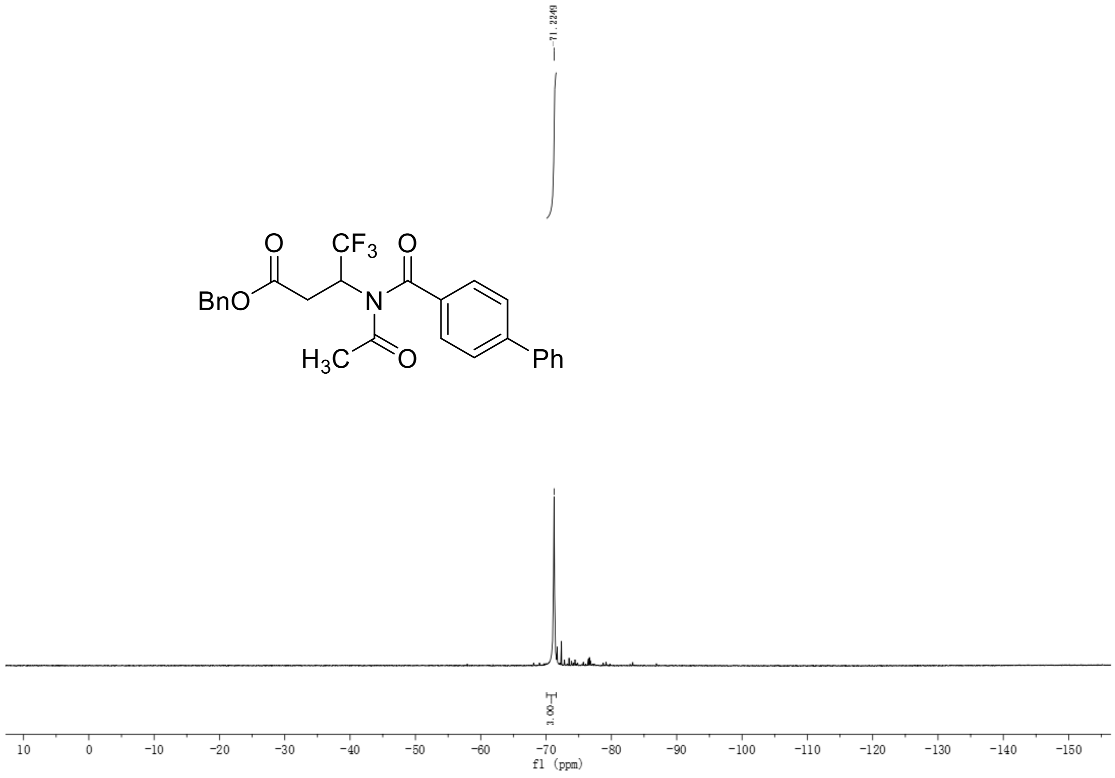


$^1H$ NMR (400 MHz, $CDCl_3$) of **4n**:

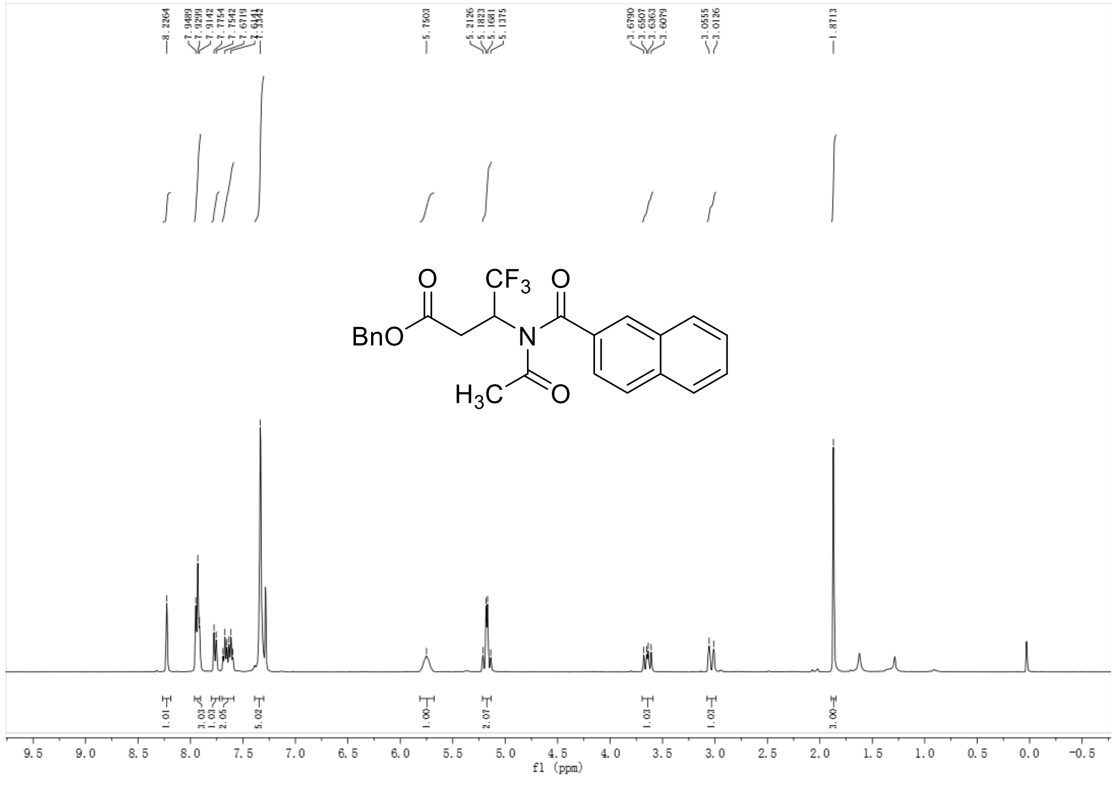

$^{13}C$ NMR (100 MHz, $CDCl_3$) of **4n**:

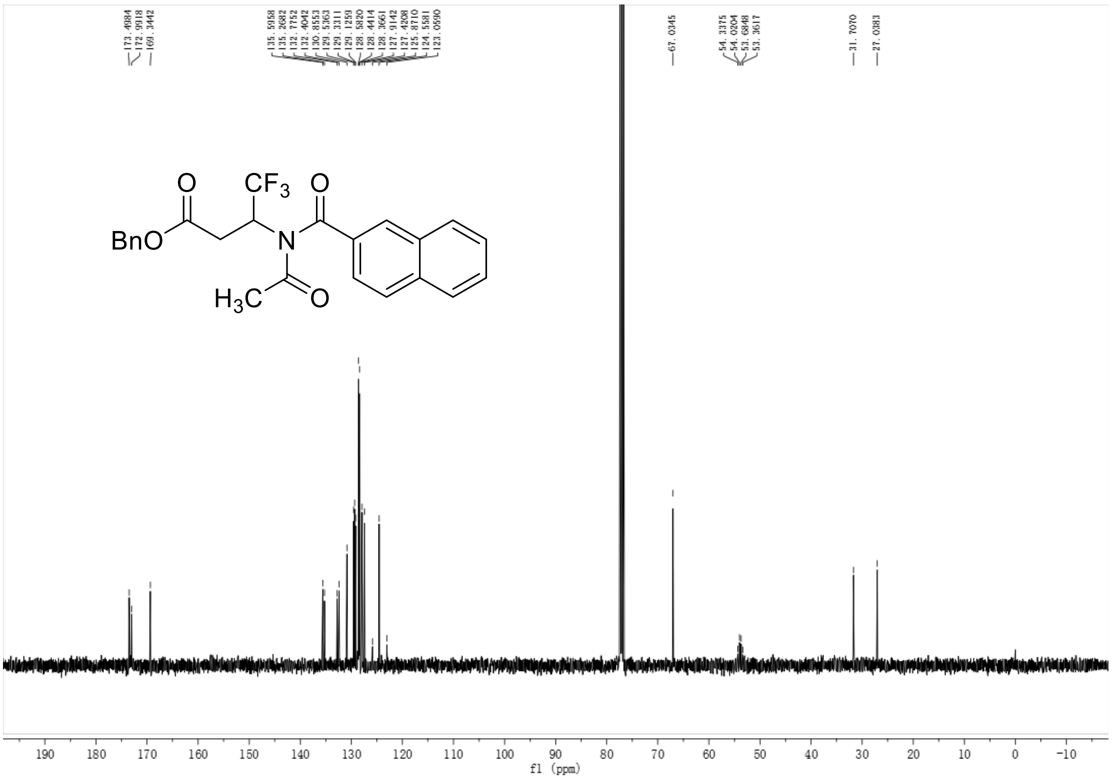

$^{19}F\{^1H\}$ NMR (376 MHz, $CDCl_3$) of **4n**:

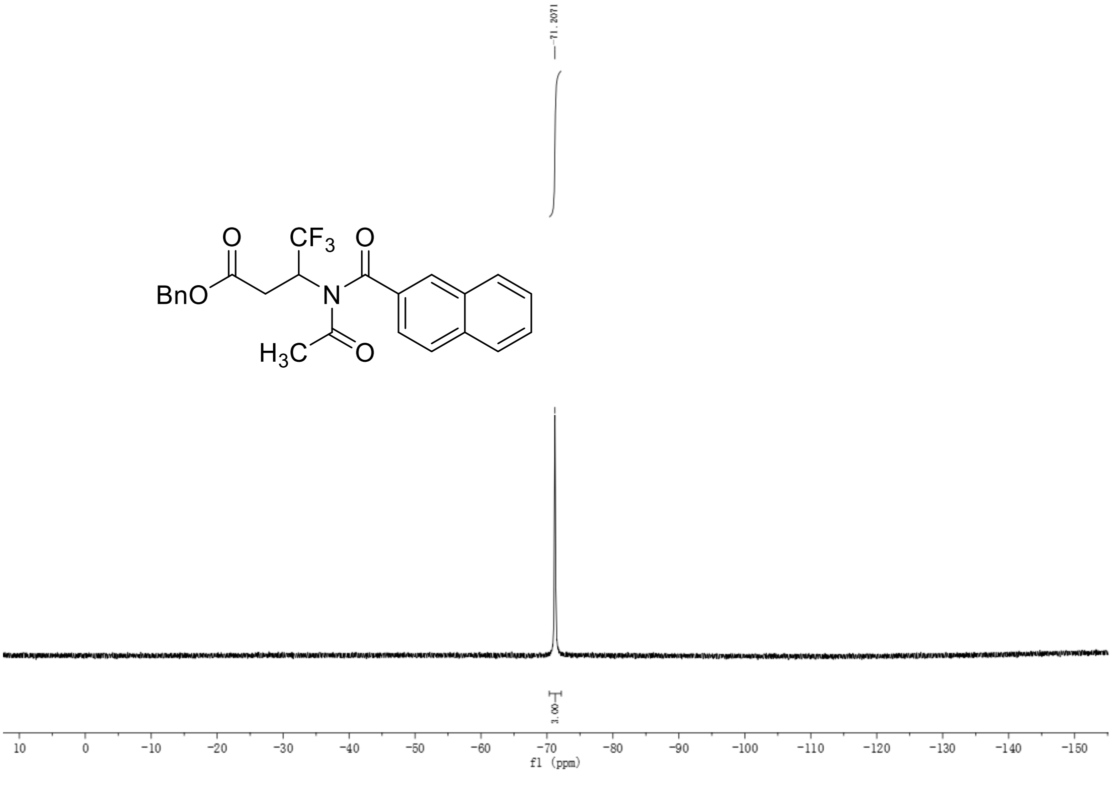

$^{1}$H NMR (400 MHz, $CDCl_3$) of **4o**:

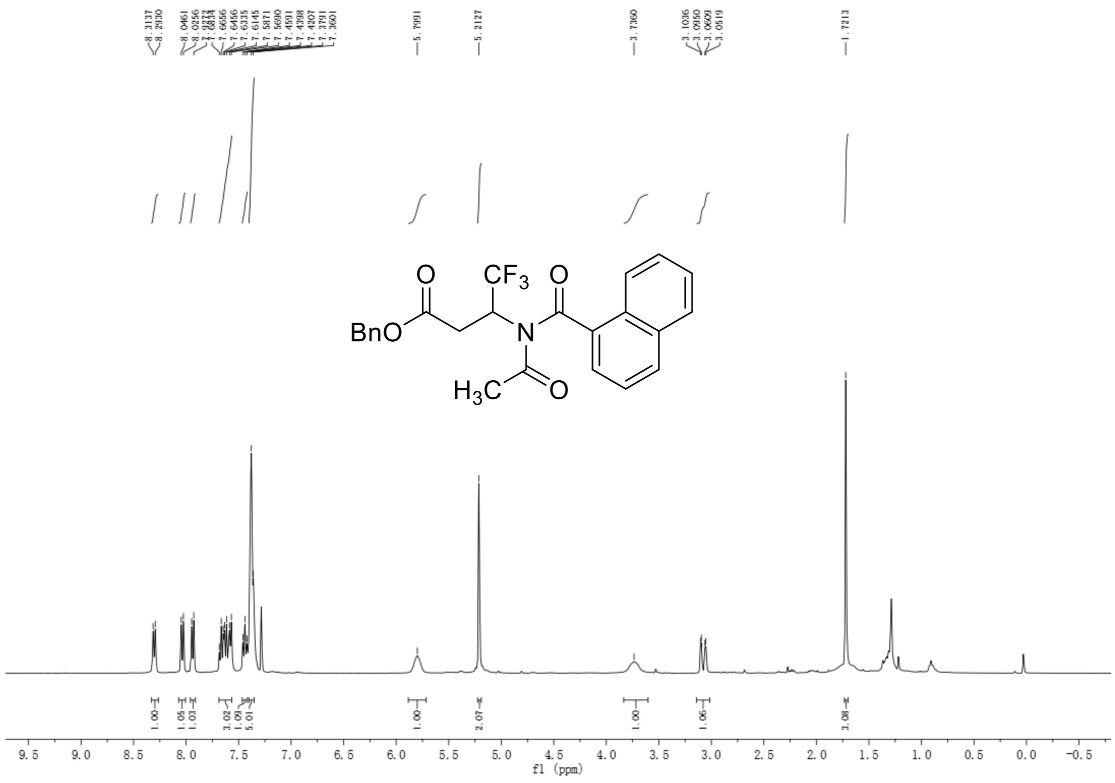

$^{13}$C NMR (100 MHz, $CDCl_3$) of **4o**:

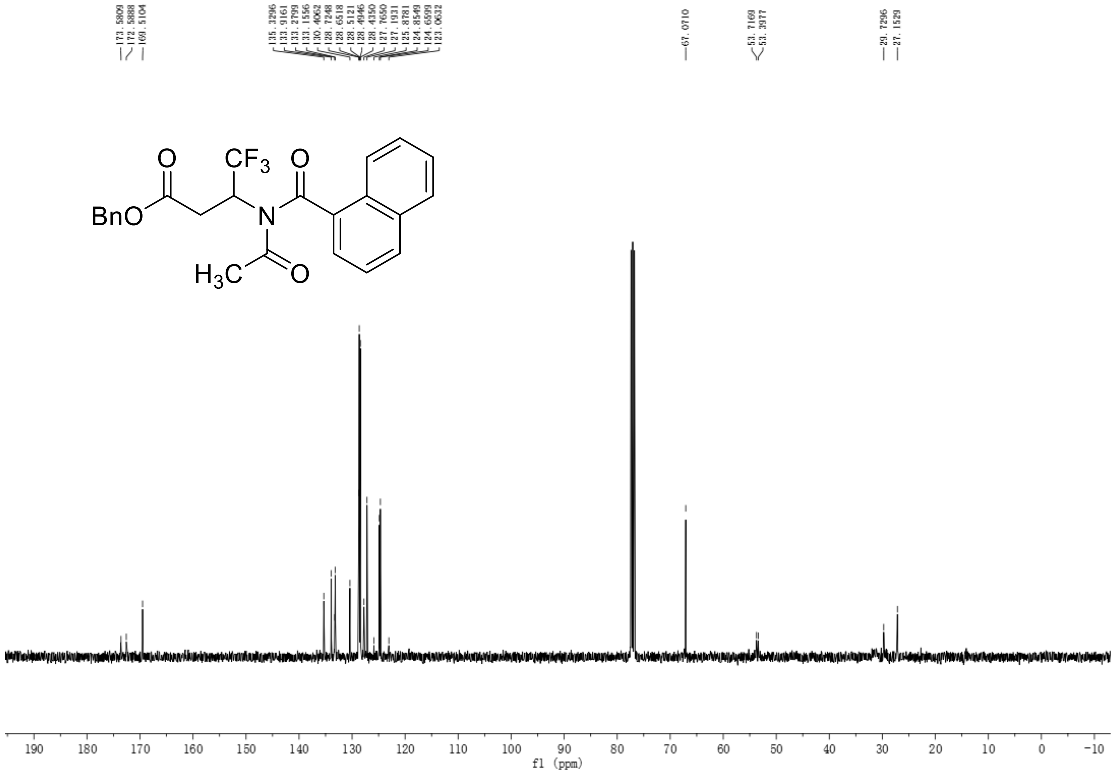

$^{19}F\{^{1}H\}$ NMR (376 MHz, $CDCl_3$) of **4o**:

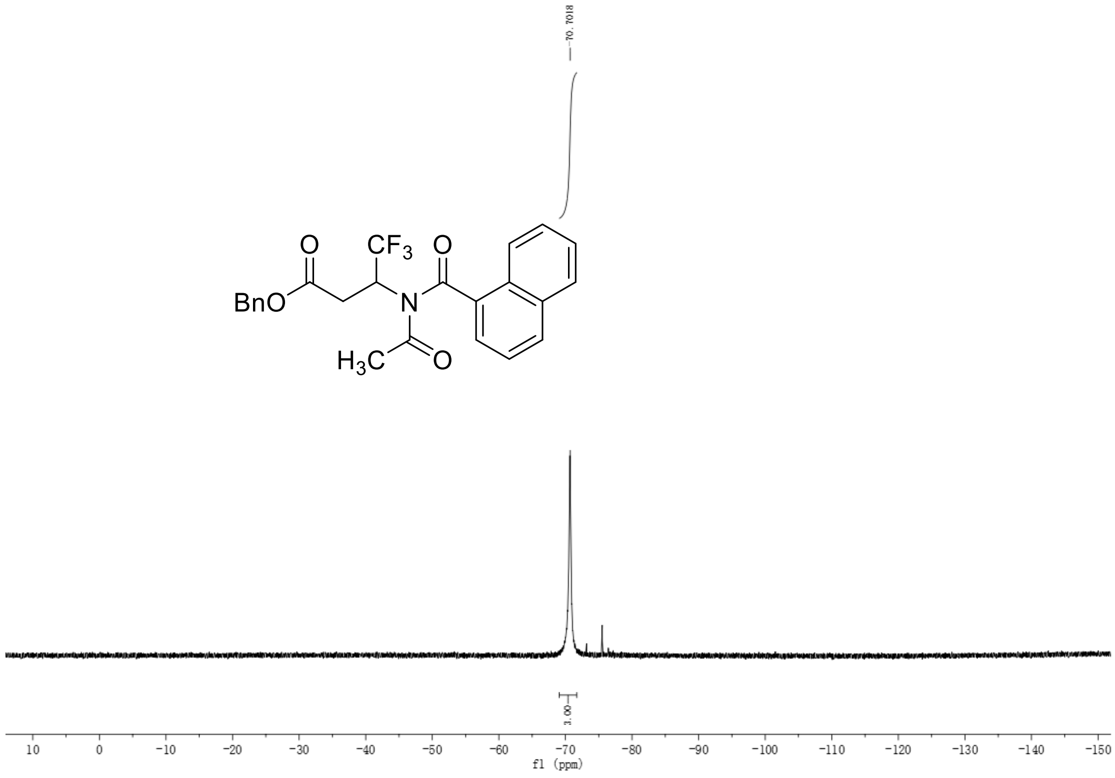

$^{1}H$ NMR (400 MHz, $CDCl_3$) of **4q**:

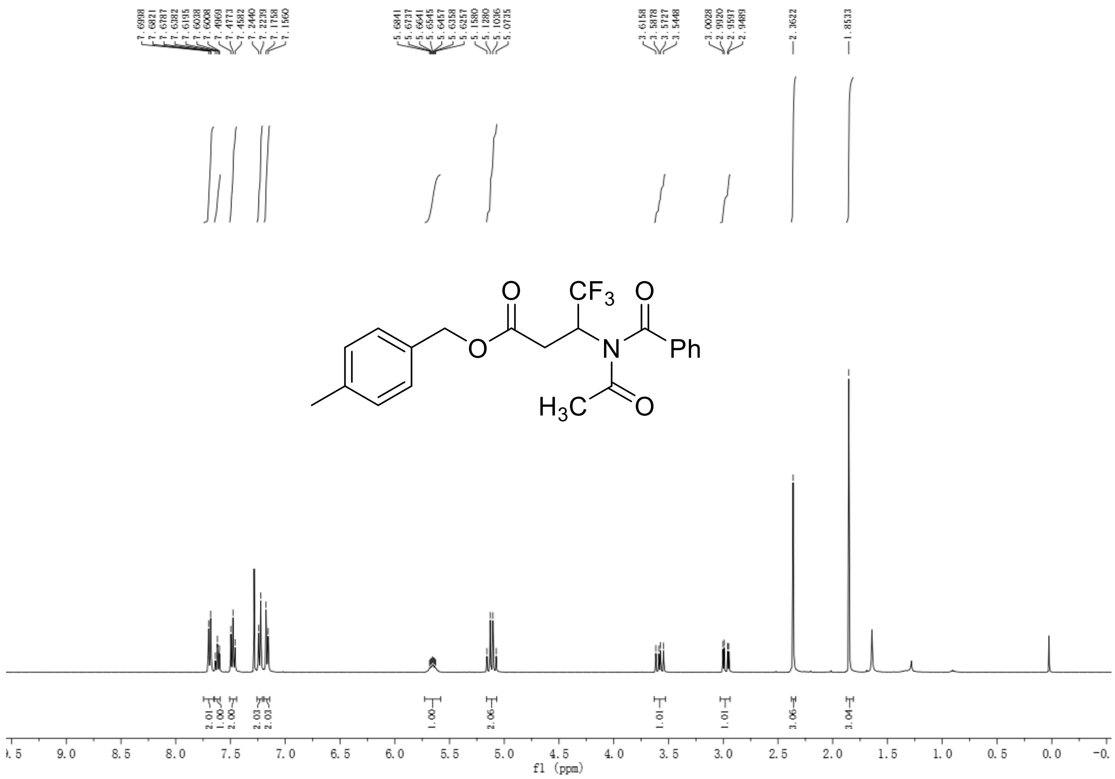

$^{13}$C NMR (100 MHz, CDCl3) of **4q**:

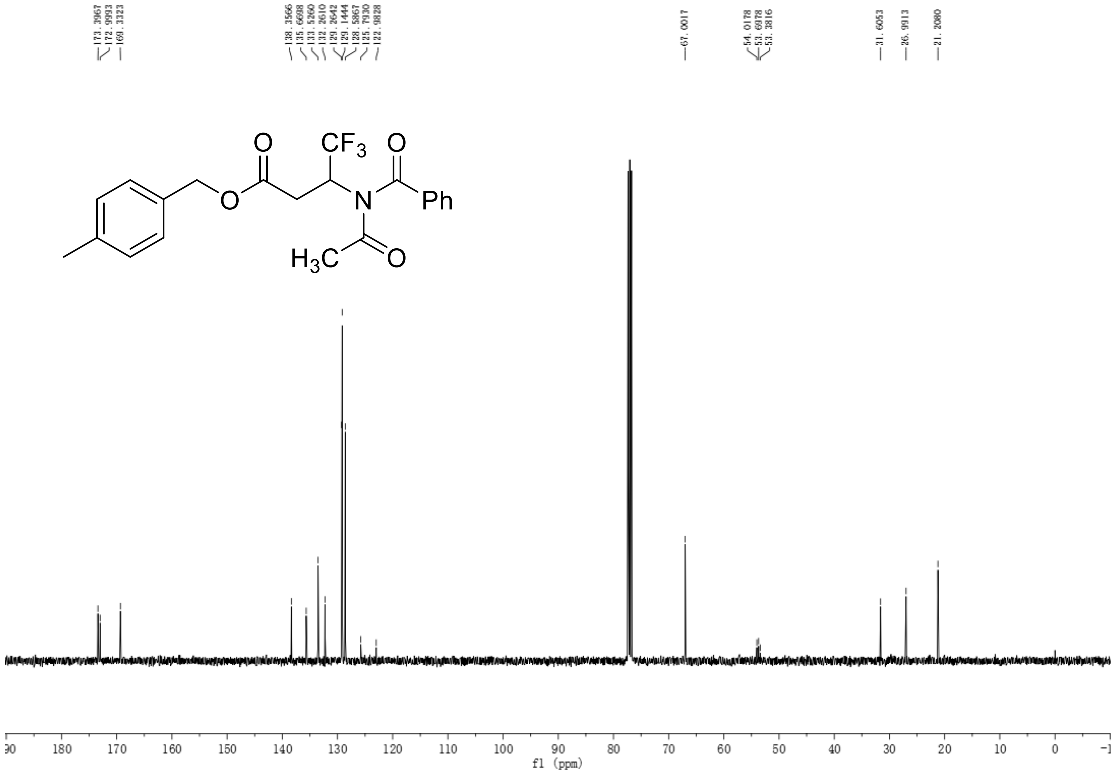

$^{19}$F{$^{1}$H} NMR (376 MHz, CDCl3) of **4q**:

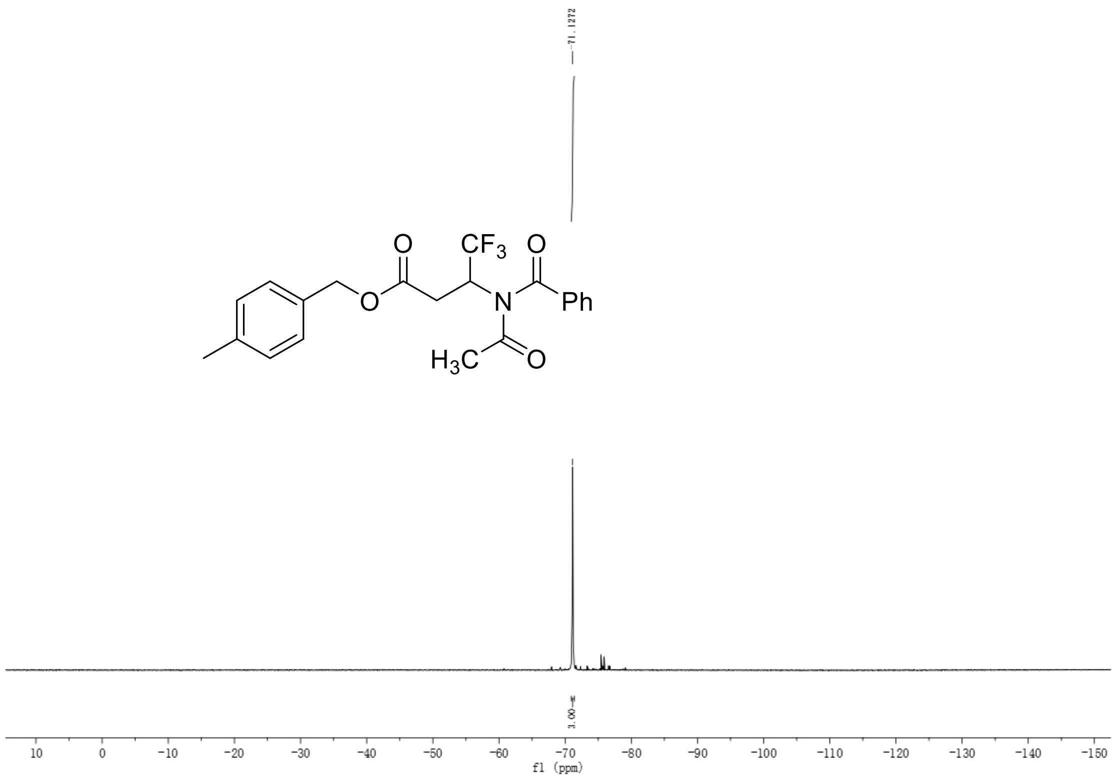

$^{1}$H NMR (400 MHz, $CDCl_3$) of **4r**:

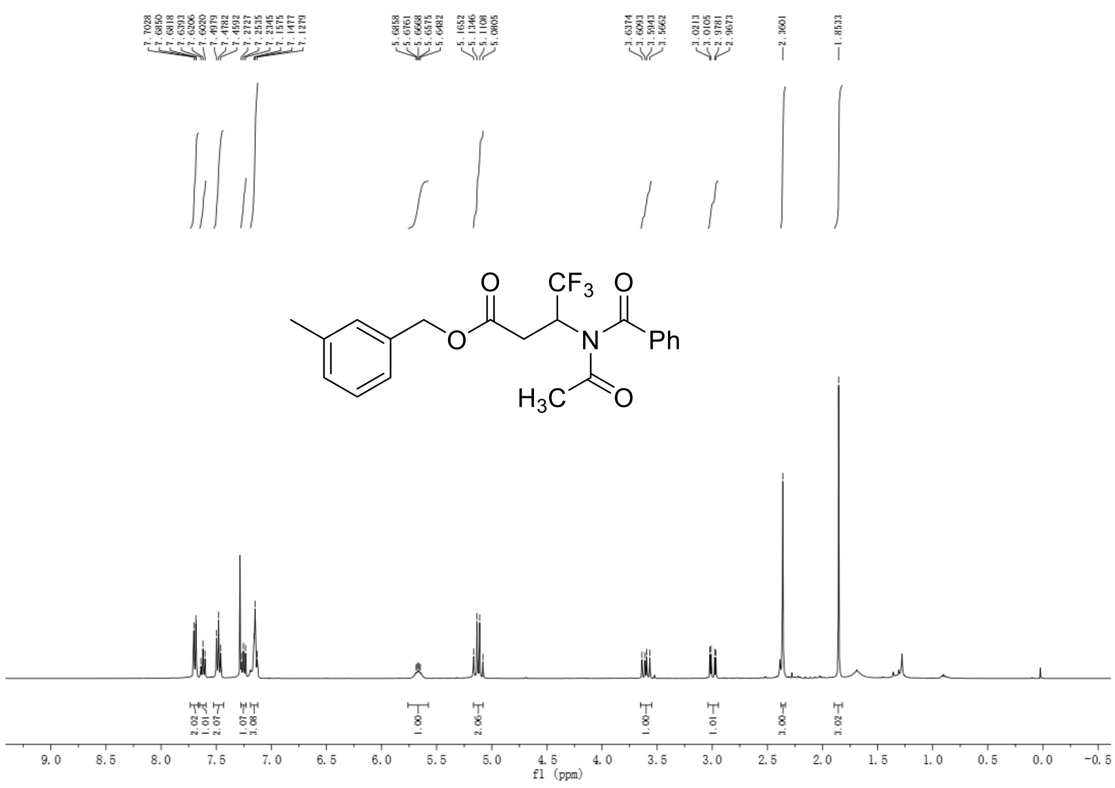

$^{13}$C NMR (100 MHz, $CDCl_3$) of **4r**:

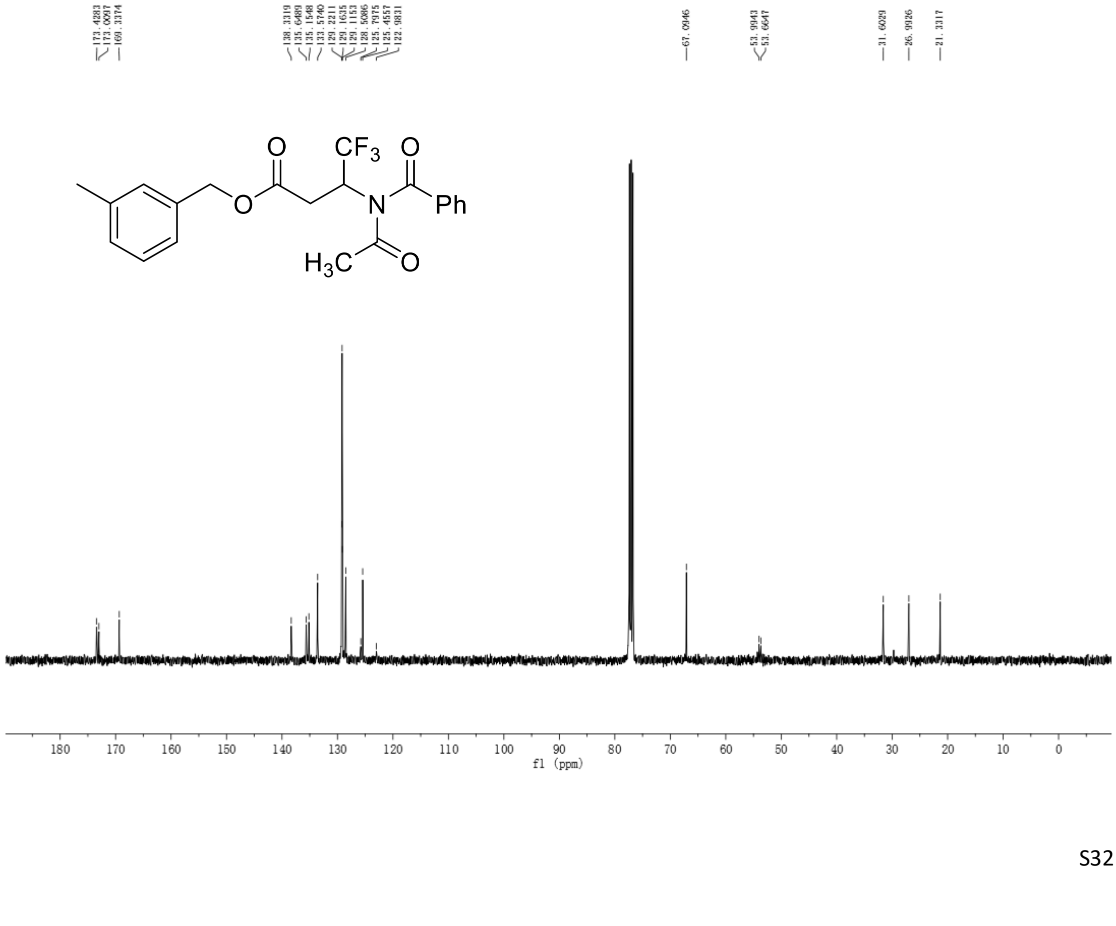

$^{19}F\{^{1}H\}$ NMR (376 MHz, $CDCl_3$) of **4r**:

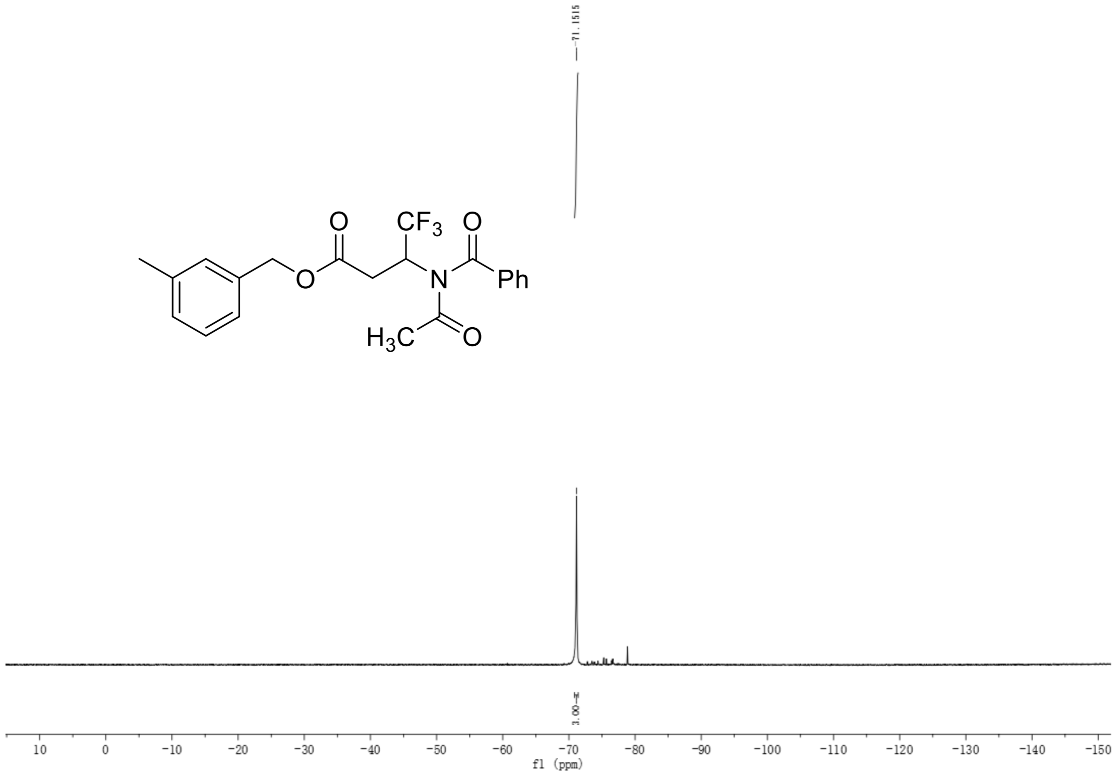


$^{1}H$ NMR (400 MHz, $CDCl_3$) of **4s**:

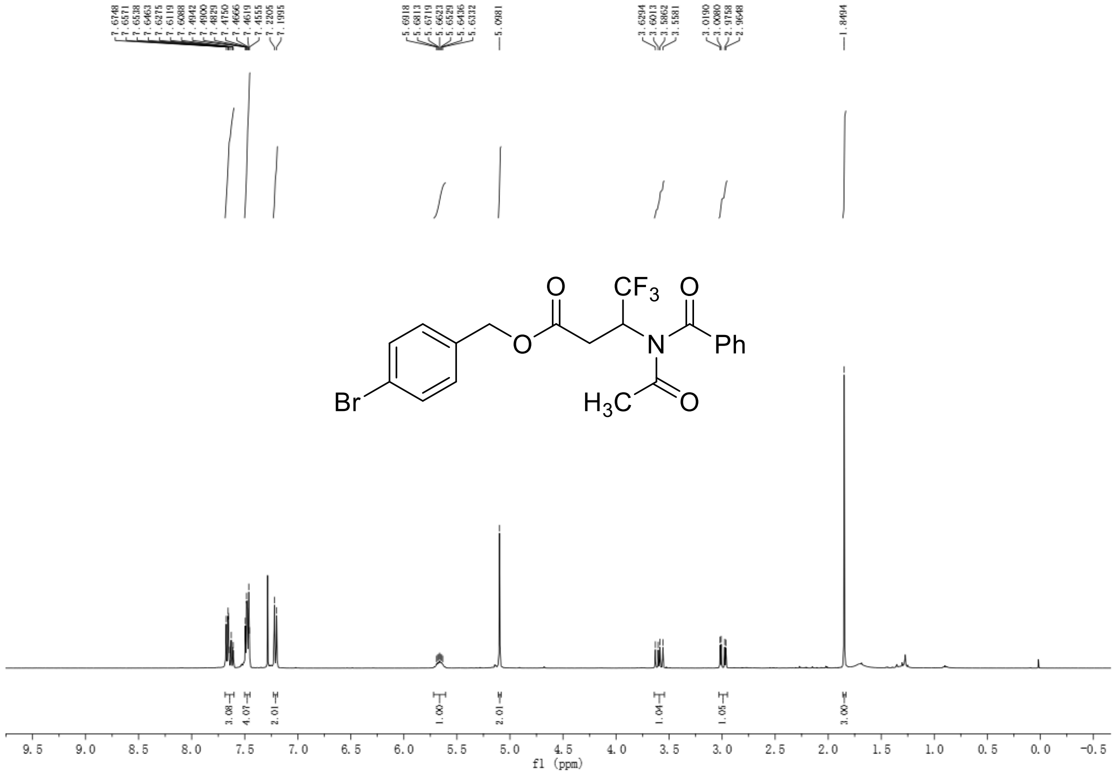

$^{13}C$ NMR (100 MHz, $CDCl_3$) of **4s**:

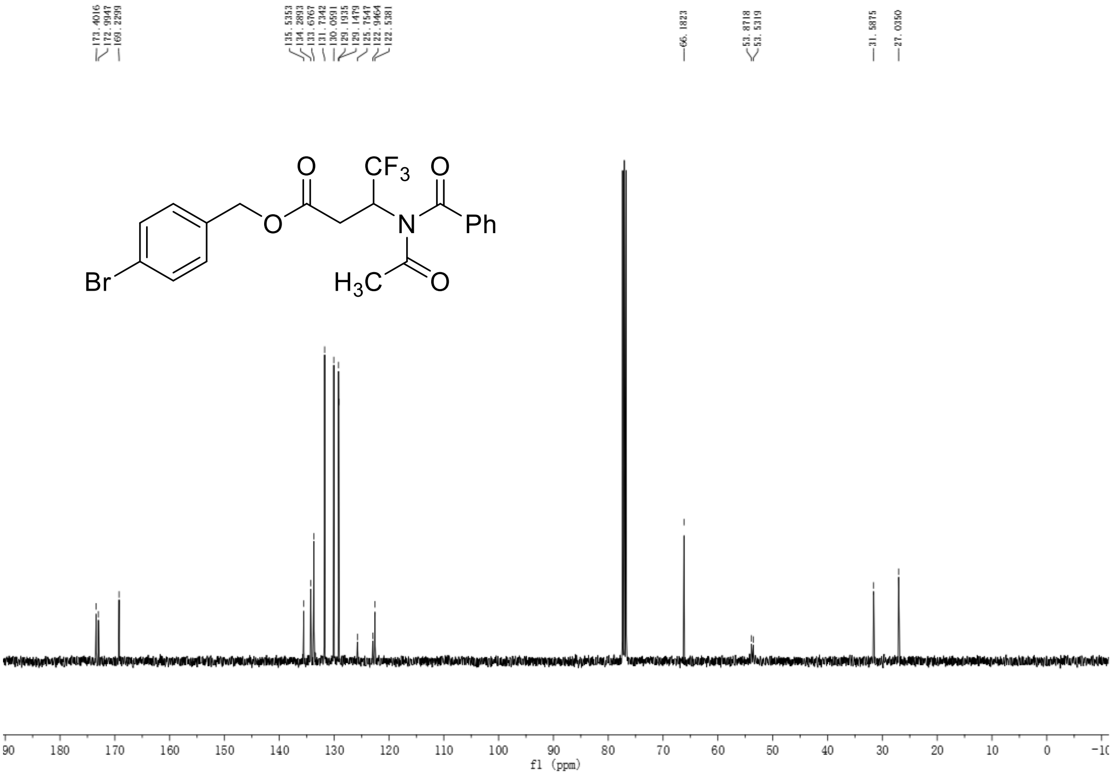

$^{19}F\{^1H\}$ NMR (376 MHz, $CDCl_3$) of **4s**:

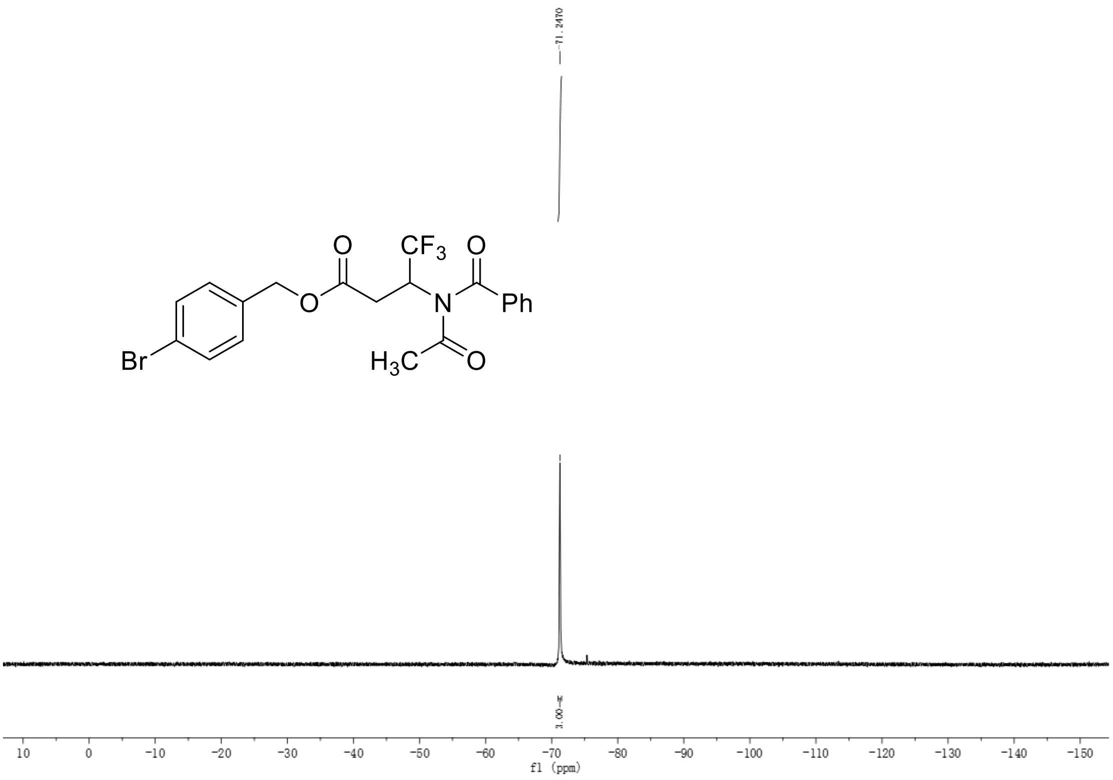

$^{1}$H NMR (400 MHz, $CDCl_3$) of **4t**:

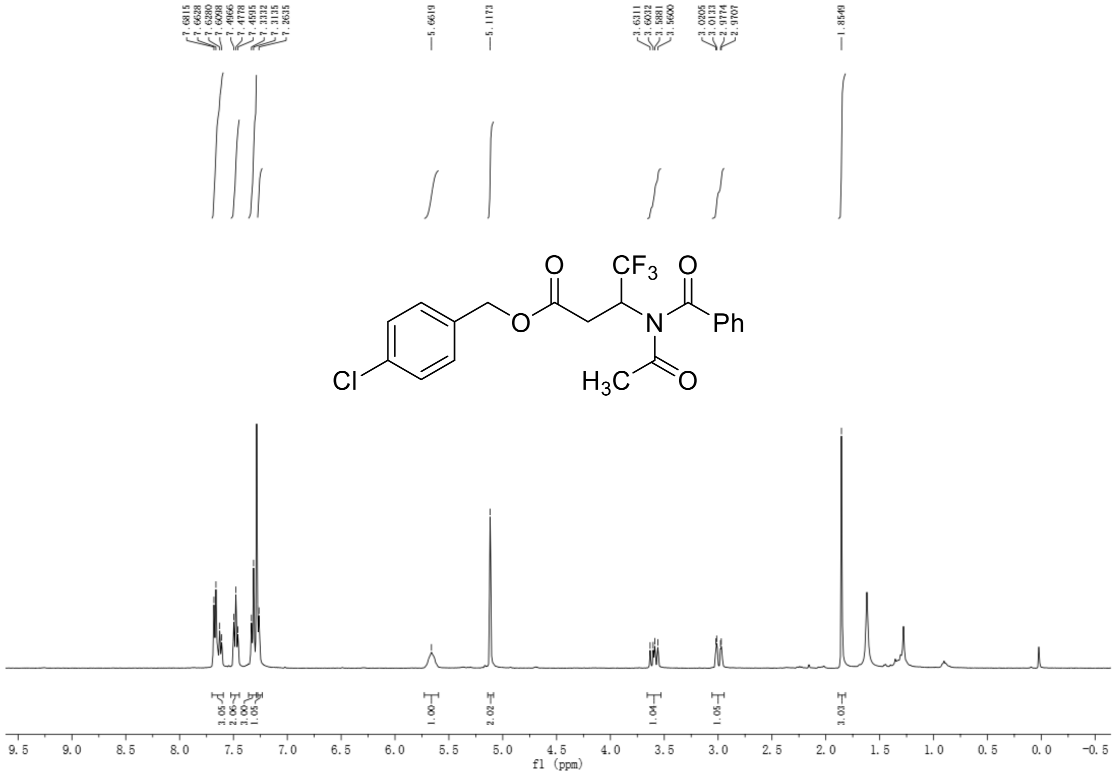


$^{13}$C NMR (100 MHz, $CDCl_3$) of **4t**:

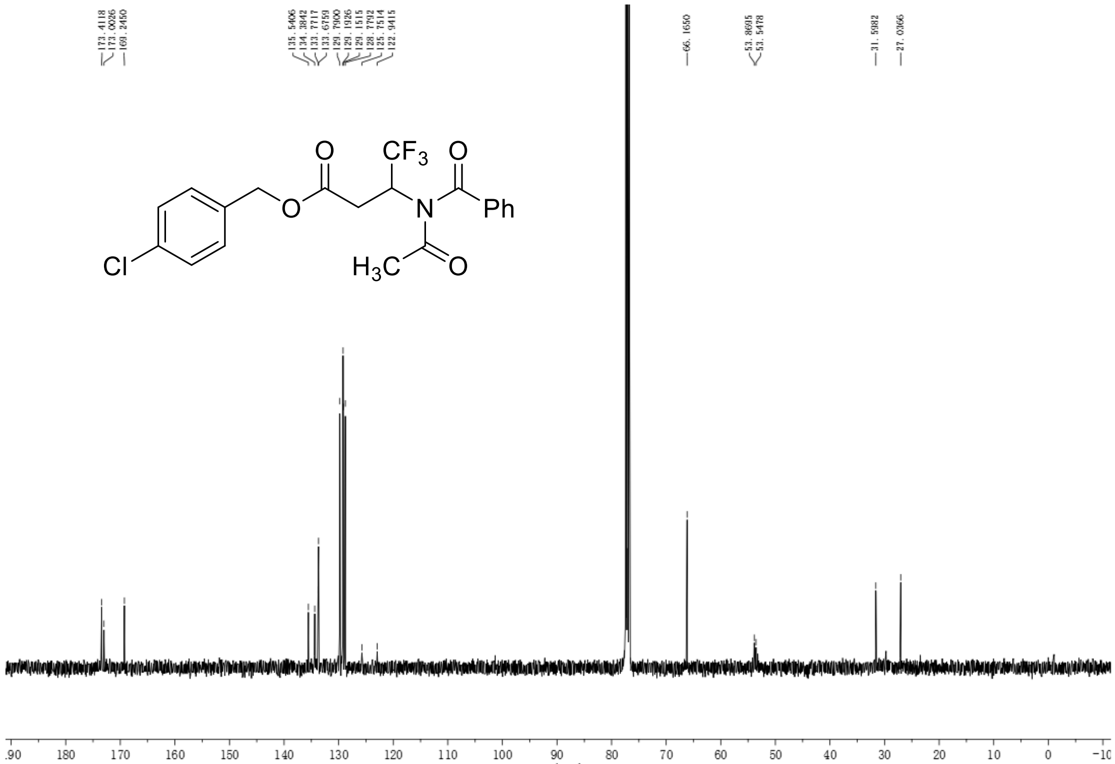

$^{19}F\{^1H\}$ NMR (376 MHz, $CDCl_3$) of **4t**:

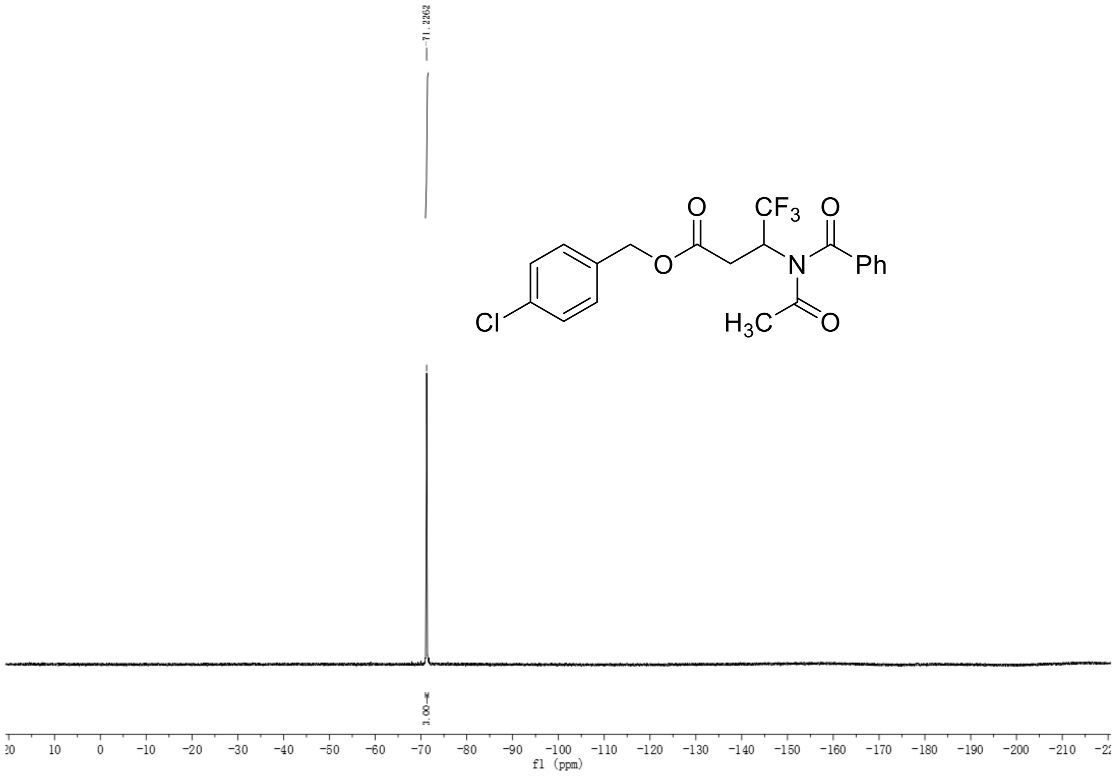

Supplement: File 1 — Experimental details and spectral data. [file Beilstein_J_Org_Chem-20-212-s001.pdf]
